# The Foreign Oligochaete Species *Quistadrilus multisetosus* (Smith, 1900) in Lake Geneva: Morphological and Molecular Characterization and Environmental Influences on Its Distribution

**DOI:** 10.3390/biology9120436

**Published:** 2020-12-01

**Authors:** Régis Vivien, Michel Lafont, Brigitte Lods-Crozet, Maria Holzmann, Laure Apothéloz-Perret-Gentil, Yaniss Guigoz, Benoit J. D. Ferrari

**Affiliations:** 1 Swiss Centre for Applied Ecotoxicology (Ecotox Centre), EPFL ENAC IIE-GE, 1015 Lausanne, Switzerland; benoit.ferrari@centreecotox.ch; 2 Univ Lyon, Université Claude Bernard Lyon 1, CNRS, ENTPE, UMR 5023 LEHNA, F-69622, Villeurbanne, France; michel.lafont33@sfr.fr; 3 Musée cantonal de zoologie, Pl. de la Riponne 6, 1005 Lausanne, Switzerland; Brigitte.Lods-Crozet@bluewin.ch; 4 Department of Genetics and Evolution, University of Geneva, Boulevard d’Ivoy 4, 1205 Geneva, Switzerland; Maria.Holzmann@unige.ch (M.H.); Laure.Perret-Gentil@unige.ch (L.A.-P.-G.); 5 ID-Gene Ecodiagnostics, Campus Biotech Innovation Park, Avenue de Sécheron 15, 1202 Geneva, Switzerland; 6 enviroSPACE, University of Geneva, Boulevard Carl-Vogt 66, 1205 Geneva, Switzerland; Yaniss.Guigoz@unige.ch

**Keywords:** oligochaete, *Quistadrilus multisetosus*, Lake Geneva, repartition, ecology, invasive potential, identification key, phylogenetic analysis

## Abstract

**Simple Summary:**

The presence of the oligochaete species *Quistadrilus multisetosus* (Smith, 1900), originating from North America, has been mentioned in Europe for some decades and was recently found in Swiss lakes. Here, we report its repartition and abundance in Lake Geneva based on morphological and eDNA surveys and study its ecology and invasive potential in this lake. We also provide an identification key of this species and two closely related species and describe the phylogenetic position of *Q. multisetosus* within several Tubificinae lineages based on the cytochrome c oxidase marker. Our results showed that this species was restricted to an area close to the outlet of a wastewater treatment plant and to a combined sewer overflow, was highly tolerant to organic matter pollution and had a limited capacity to disseminate in this lake. Even if the trophic status (oligo-mesotrophic) of Lake Geneva seems unfavorable for the development of this species, we recommend continuing monitoring its presence in this lake in the future, as the current warming of waters could contribute to its expansion.

**Abstract:**

The presence of the oligochaete species *Quistadrilus multisetosus* (Smith, 1900) originating from North America has been mentioned for several decades in Europe, the Middle East and Russia. Its distribution and abundance in Europe is still unknown but it can be considered as potentially invasive. This species was recently discovered in Lake Geneva (Switzerland/France) and three other Swiss lakes. The aims of the present work are to report its repartition and abundance in Lake Geneva, to study its ecology and to determine its invasive potential in this lake. We also provide an identification key for correctly differentiating *Q. multisetosus* from the closely related species *Spirosperma ferox* Eisen, 1879 and *Embolocephalus velutinus* (Grube, 1879), and study the phylogenetic position of *Q. multisetosus* within several Tubificinae lineages based on the cytochrome c oxidase (COI) marker. Twenty-eight sites have been monitored since 2009 in Lake Geneva. In several sites, the COI sequence corresponding to this species was also searched for in sediment samples using high-throughput sequencing. In addition, we examined specimens collected in this lake before 2009 likely to belong to *Q. multisetosus* and to have been misidentified. We found that *Q. multisetosus* was only present in the lake downstream of a wastewater treatment plant and a combined sewer overflow in the Vidy Bay (near Lausanne) and at a site located nearby. These results confirmed the high tolerance of this species to organic matter pollution. *Q. multisetosus* was already present in this location in 1974 (misidentified as *Spirosperma ferox*), which suggests that *Q. multisetosus* has a limited capacity to disseminate in this lake. However, we recommend continuing monitoring its presence in Lake Geneva in the future, especially in the context of warming of waters that could contribute to the expansion of this species.

## 1. Introduction

*Quistadrilus multisetosus* (Smith, 1900) is a common aquatic oligochaete species in North America [1,2]. Its presence has been mentioned for several decades in waterbodies in some European countries, in the Middle East and in Russia [3,4,5,6,7]. This species has probably been in Europe for a long time. Indeed, the species *Peloscolex moszynskii*, described by Kasprzak in Poland in 1971 [8], is a synonym of *Quistadrilus multisetosus* [3]. The real distribution of *Q. multisetosus* in Europe is not precisely known. So far, its presence in Europe was mentioned in a relatively small number of localities but is certainly underestimated. *Q. multisetosus* can be confounded with some other Tubificinae and its occurrence is not routinely monitored. In Switzerland, this species has only been mentioned in Lake Biel (one specimen on the shore, [9]), in Lake Lucerne (one specimen on the shore, unpublished data) and in lake Constance [10].

*Quistadrilus multisetosus* is recognizable by the presence of prominent light sensory papillae arranged in a transversal row in every segment on the chaetal line, by the presence of foreign particles irregularly arranged in some parts of the body and by characteristic ventral and dorsal chaetae [11,12,13]. The species can be confounded with two other Tubificinae species, *Embolocephalus velutinus* (Grube, 1879) and *Spirosperma ferox* Eisen, 1879 also covered by foreign particles and especially *S. ferox* that has a similar shape of chaetae.

In the present work, we mention the presence of *Quistadrilus multisetosus* in Lake Geneva, report its current distribution and abundance in this lake, complement the existing data concerning its ecology and determine the invasive potential of this species in this lake. Twenty-eight sites have been investigated in Lake Geneva since 2009, principally along the shores. One hundred to 427 oligochaete specimens were identified morphologically per site and at several sites, we genetically searched for the COI sequence of *Q. multisetosus* in sediment samples using high-throughput sequencing (HTS). Besides, we examined specimens collected in this lake before 2009 likely to belong to *Q. multisetosus* and to have been misidentified. In addition, a revision of the morphological criteria, including newly observed ones, enabling to differentiate between *Q. multisetosus*, *Spirosperma ferox* and *Embolocephalus velutinus* is performed and an identification key is provided. Finally, we present the phylogenetic position of *Q. multisetosus* within several Tubificinae lineages found in Switzerland based on COI analysis and check the genetic divergence between *Q. multisetosus* and closely related species.

## 2. Materials and Methods

### 2.1. Sites and Repartition of the Analyses

**Figure 1:**
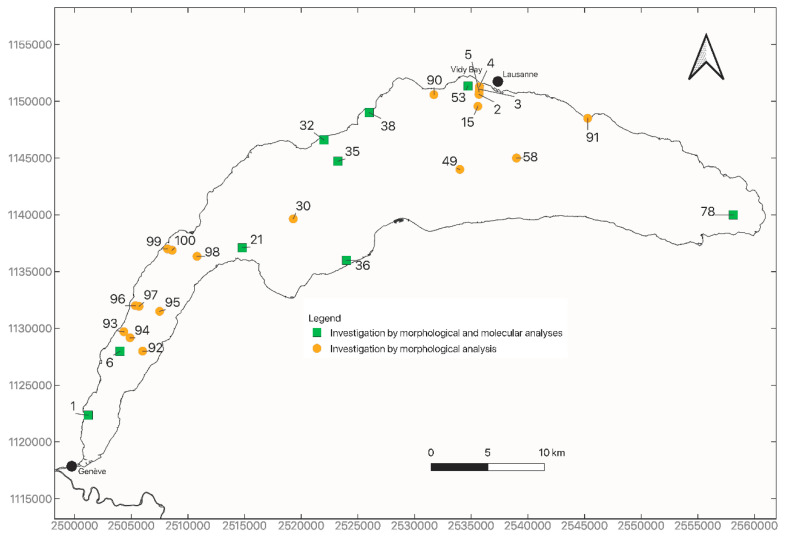
Map of all the studied sites in Lake Geneva, with indication of the analyses (morphology, genetic) performed per site.

Twenty-eight sites were studied in Lake Geneva between 2009 and 2019 [14,15,16] (Figure 1, Table 1). Twenty-three sites had a sampling depth between 10 m and 80 m and 5 sites between 149 m and 309 m. One campaign was performed at all sites except one site (site 32, 2015 and 2017). Sites 2, 3, 4, 5, 15 and 53 are located in the Vidy Bay. Site 53 is very close to the outlet of the wastewater treatment plant (WWTP) of the city of Lausanne and is thus strongly impacted by its effluents. Sites 2, 3, 4, 5 and 15 are under the influence of both this WWTP and a combined sewer overflow (CSO). These sites are on a transect aligned with the CSO from 24 m deep (Site 4) to 188 m deep (Site 15). Among these five sites, the most impacted by the effluents from the WWTP and CSO are sites 3 to 5, sites 2 and 15 being located farther and deeper. Sediments of the Vidy Bay contain particularly high concentrations of organic matter, metals, PCBs and PAHs [17]. A morphological analysis of sampled oligochaetes was performed on all 28 sites. In addition, genetic analyses of sediment samples (HTS) were performed to detect the presence of *Q. multisetosus* at 9 sites (1, 32, 53, 78, 6, 21, 36, 35 and 38), among them one site (32) at two different times.

### 2.2. Sampling

Sediment samples (3 L) were collected using an Ekman type grab sampler. At each site, 3 or 5 subsamples (one sample every 10–20 m) were collected (Table 1). For the sites studied in 2009 and 2015, the 3 or 5 subsamples were treated individually, while for the sites studied between 2016 and 2019, the 3 subsamples were combined. For each of the sites 1, 32, 53, 78, 6, 21, 36, 35 and 38, a composite sample of sediments was first collected with a spoon for the HTS analyses by transferring 10 mL of sediment per grab sampler to a unique 50 mL tube (i.e., the 3 or 5 subsamples were mixed). The 50 mL tubes were then preserved at 4 °C during collection and frozen at −20 °C once back at the laboratory. The sediment was fixed in the field with 20% neutral buffered formalin or 37% low-pH formalin (ThermoFisher Scientific, Ecublens, Switzerland) and adjusted to a final formaldehyde concentration of 4%. Back at the laboratory, sediment samples were sieved at 0.5 mm or 0.315 mm mesh size. The retained material was transferred to a plastic box and preserved in absolute ethanol at −20 °C or in formalin 4% at 4 °C.

### 2.3. Morphological Examination of Oligochaete Communities

For each sediment sample, the material retained in the sieve was placed in a subsampling square box (5 × 5 cells), and the contents of randomly selected cells were transferred into a Petri dish and examined under a stereomicroscope until 100 or 120 specimens were collected. Sorted specimens were then mounted on slides in a coating solution composed of lactic acid, glycerol and polyvinylic alcohol [18]. Oligochaete specimens were identified to the lowest practical level (species if possible) using a compound microscope. In total, between 100 and 467 specimens were identified per site (Table 1).

### 2.4. Examination of Specimens from Collections

We examined some oligochaete specimens identified as *Spirosperma ferox* collected in 1974 in Lake Geneva in the Vidy Bay [19]. As *S. ferox* was described in lakes as sensitive to moderately sensitive to pollution by organic matter [20], we suspected that these specimens had been misidentified and belonged in fact to *Quistadrilus multisetosus*. Oligochaetes had been collected in many sites in this area, mainly located downstream of the discharges from the WWTP of the city of Lausanne and directly or potentially impacted by its effluents.

### 2.5. Genetic Analyses

Identification of organisms is possible by sequencing a short DNA sequence (called DNA barcode) that is similar or very close between individuals of the same species. The mitochondrial COI barcode was suggested for identification of aquatic and terrestrial oligochaetes and a 10% threshold of COI divergence has been considered appropriate for distinguishing between most aquatic oligochaete species [21,22,23,24]. eDNA metabacoding is a recently developed technology enabling the ability to sequence all species present in an environmental sample (water, sediments, etc.) [25]. It is used for diverse purposes, including invasive species detection [26,27], the establishment of inventories of species [28,29] and assessment of the biological quality of ecosystems [30].

#### 2.5.1. Acquisition of the COI Barcode of *Quistadrilus Multisetosus*

Three *Quistadrilus multisetosus* specimens collected at Site 53 were individually analyzed to obtain the sequence of a fragment of 658 pb of the COI gene. Total genomic DNA was extracted from tissue samples using the guanidine thiocyanate method described by Tkach and Pawlowski [31]. A 658 base pairs fragment of the COI gene was amplified using primers LCO 1490 and HCO 2198 [32]. PCR amplifications were performed in a total volume of 20 μL containing 0.2 μL of Taq polymerase 5 U/μL (Roche, Basel, Switzerland), 2 μL of the PCR buffer (10× concentrated) with MgCl_2_ (Roche), 0.5 μL of each primer (10 μM each), 0.4 μL of a mix containing 10 mM of each dNTP (Roche) and 1 μL of DNA template. The PCR comprised an initial denaturation step at 95 °C for 5 min, followed by 35 cycles of denaturation at 95 °C for 40 s, annealing at 44 °C for 45 s and elongation at 72 °C for 1 min and a final elongation step at 72 °C for 8 min. The PCR products were then directly and bi-directionally Sanger sequenced on an ABI 3031 automated sequencer (Applied Biosystems, Foster City, CA, USA) using the same primers as above and following the manufacturer’s protocol. The raw sequence editing and the generation of contiguous sequences were performed using CodonCode Aligner (CodonCode Corporation, Centerville, OH, USA). The obtained COI sequences of *Q. multisetosus* are deposited in the European Nucleotide Archive.

#### 2.5.2. Construction of a COI Phylogenetic Tree

The obtained sequences of *Q. multisetosus* were added to a database including Tubificinae lineages found in Switzerland [23] using the Muscle automatic alignment option as implemented in SeaView vs. 4.3.3. [33]. The alignment contains 35 sequences with 658 sites of which 351 are without polymorphism. Nucleotide frequencies are 0.37 (A), 0.21 (C), 0.10 (G) and 0.32 (T). A phylogenetic tree was constructed using maximum likelihood phylogeny (PhyML 3.0) as implemented in ATGC: PhyML [34]. An automatic model selection by SMS [35] based on Akaike Information Criterion (AIC) was used yielding in a GTR + G + I substitution model being selected for the analysis. The initial tree is based on BioNJ. An additional tree was constructed using FastMe 2.0, a distance-based phylogeny inference program as implemented in ATGC: FastMe [36]. F84 was used as substitution model with gamma distributed rates across sites and tree refinement with Subtree Pruning and Regrafting (SPR). Bootstrap values (BV) are based on 100 replicates for PhyML and FastMe analyses. A 10% threshold of COI divergence was applied to distinguish between species (species = lineage) (cf. Section 2.5). The intra- and inter-lineage distances were calculated using the K2P model in MEGA 5.1 [37].

#### 2.5.3. eDNA Metabarcoding

##### DNA Extraction, PCR Amplification, Library Preparation and Illumina Sequencing

Total genomic DNA was extracted from the total sediment samples using the DNeasy PowerMax Soil Kit (Qiagen, Hilden, Germany) following the manufacturer’s protocol.

A COI fragment (313 base pairs) was amplified using the primers specific to metazoans “mlCOIintF” and “jgHCO2198” [38]. PCR amplifications were performed exactly as described in Section 2.5.1. Three PCR amplifications of each sample were performed. The metazoan primers were tagged by bearing eight nucleotides attached at each primer’s 5′ extremity. A unique combination of tagged primers was used for each sample in order to multiplex all samples in a unique sequencing library [39]. Pools of the three PCR replicates were then quantified with capillary electrophoresis using QIAxcel instrument (Qiagen, Hilden, Germany). Equimolar concentrations of PCR products were pooled into a single tube that was purified using High Pure PCR Product Purification kit (Roche Diagnostics, Risch-Rotkreuz, Switzerland). The library preparation was performed using a TruSeq^®^ DNA PCR-Free Library Preparation Kit (Illumina, San Diego, CA, USA) and was quantified with qPCR using KAPA Library Quantification Kit (Roche). Finally, the library was sequenced on a MiSeq instrument using paired-end sequencing for 500 cycles with Standard kit v2. Raw sequences of the 10 samples are accessible in the Short Read Archive under the BioProject number PRJNA678609.

##### Sequence Analysis

Bioinformatics analyses were performed using the web application SLIM [40]. Raw fastq reads were first demultiplexed using the dtd algorithm implemented in SLIM. Then, they were quality-filtered by removing any sequence with a mean quality score of 30 and also removing all sequences with ambiguous bases or any mismatch in the tagged primer. Paired-end reads were then assembled using simple bayesian algorithm implemented in pandaseq [41]. Chimera removing and the OTUs clustering at 97% was performed using vsearch [42].

All the sequences were taxonomically assigned using the assignment function of vsearch tool [42] against a local COI oligochaete database [23], to which we had added the COI sequences of *Quistadrilus multisetosus* obtained during the present work. The sequences of our Swiss database are deposited in the European Nucleotide Archive and directly available in Vivien et al. [23] (Supplemental Files). The sequences diverging by less than 10% (in COI) were considered as belonging to the same species (cf. Section 2.5).

## 3. Results

### 3.1. Distribution and Abundance of Quistadrilus Multisetosus

**Figure 2:**
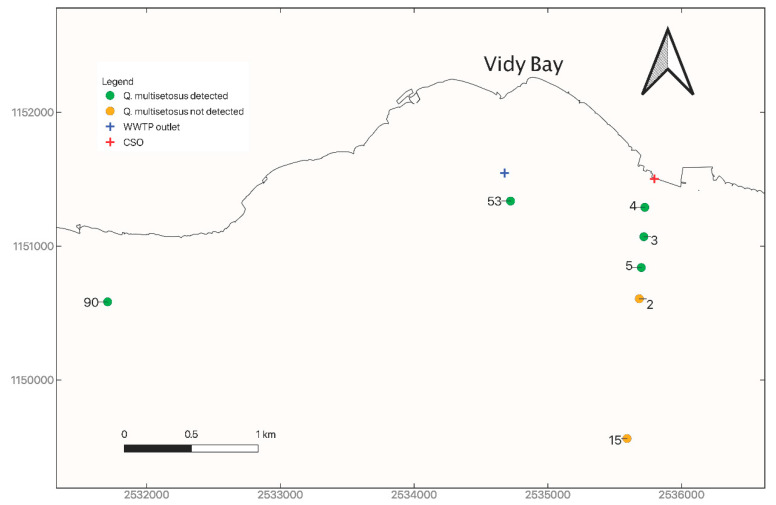
Map showing the presence/absence of *Quistadrilus multisetosus* at sites 90, 53, 4, 3, 5, 2 and 15. These results are based on both morphological and HTS analyses (concordant results). The wastewater treatment plant (WWTP) outlet and the combined sewer overflow (CSO) are indicated on the map.

*Quistadrilus multisetous* was found morphologically in quite high abundance at sites 53, 4, 3 and 5 (respectively 13%, 8%, 32% and 12%) that are all located in the Vidy Bay (Figure 2, Supplementary Tables S1 and S2). Interestingly, we observed in the transect (sites 4, 3, 5, 2 and 15, Vidy Bay) that the species was present at depths up to 60 m (Site 5), and not at 76 m (Site 2) and 188 m (Site 15) deep, although the distance between sites at 60 and 76 m depths was short (about 200 m). The species was also present but in low abundance (site 90, 2%) near the Vidy Bay, at 3.5 km to the West. No specimens of *Q. multisetosus* were found at the 23 other sites.

Concerning the HTS analyses, the percentages of reads corresponding to oligochaetes lineages were between 0.8% and 33.1% (mean = 7.8%, median = 5.6%) (Supplementary Table S3). The absence of *Quistadrilus multisetosus* was confirmed genetically at 8 of the 23 sites, as no trace of DNA of this species was found (Figure 2, Supplementary Table S3). At site 53, as expected, the genetic analyses detected the presence of *Q. multisetosus* in a high abundance, about 30% of all oligochaete reads corresponding to this species.

### 3.2. Morphological Differentiation of Quistadrilus Multisetosus from Spirosperma Ferox and Embolocephalus Velutinus

*Quistadrilus multisetosus*, *Spirosperma ferox* and *Embolocephalus velutinus* can be easily discriminated from the other tubificids with hair setae by the form of the chaetae. The presence of dark particle aggregates on their body surface is also characteristic of these three species and can be used for differentiating them from the other tubificids. However, we found in Lake Geneva one specimen of *S. ferox* without any dark particle aggregate on the body surface and *Q. multisetosus* can present few or not well visible particle aggregates. Therefore, the form of the chaeteae is determinant and should always be considered. Two of these species (*Q. multisetosus* and *E. velutinus*) have also prominent light sensory papillae arranged in a transversal row in each segment on the chaetal line but these papillae are not always well visible on fixed specimens. These papillae are certainly more visible on live specimens.

We propose below an identification key for differentiating *Quistadrilus multisetosus*, *Spirosperma ferox* and *Embolocephalus velutinus*. Several differential characters reported here are based on our own observations. The three species can be differentiated from each other by considering the following characters: presence/absence of prominent light sensory papillae, size and importance of cover of dark particle aggregates on the body surface and shape of the ventral and dorsal chaetae and of the prostomium.

The prostomium of *Embolocephalus velutinus* and *Spirosperma ferox* appears, contrarily to *Quistadrilus multisetosus*, almost always flattened. This could be explained by a retraction of the prostomium in these two species caused by the fixation step, as we could observe one specimen of *S. ferox* with a slightly elongated prostomium. We suggest the large dark and roundish formations observed in the three species and especially in *E. velutinus* and *S. ferox* are, like the small dark formations arranged in transversal rows in *Q. multisetosus* and *S. ferox*, aggregates of foreign particles due to the mucus secreted by the body surface. Indeed, we could sometimes observe on our preparations detachments of these large dark formations from the body. The retention of foreign particles by mucus secreted by the oligochaete body surface is well known [11], but the mechanism of formation of such large and roundish structures seems to have not been the object of any research. *E. velutinus* presents, like *S. ferox* and *Q. multisetosus*, small dark particle aggregates. Indeed, we found one specimen of *E. velutinus* in Lake Geneva without any large dark and roundish particle aggregates and this specimen presented clearly these small dark particle aggregates arranged in transversal lines in some parts of the body surface.

**Figure 3:**
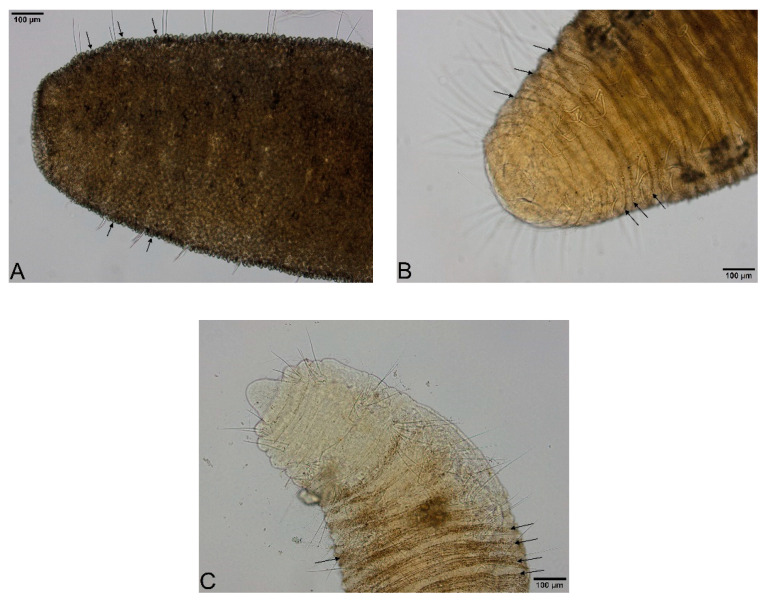
(**A**): Anterior part of *Embolocephalus velutinus*. (**B**): Anterior part of *Spirosperma ferox*. (**C**): Anterior part of *Quistadrilus multisetosus*. The arrows indicate some large dark and roundish particle aggregates in A and some transversal lines of small dark particle aggregates in B and C. Author: Régis Vivien.

**Figure 4:**
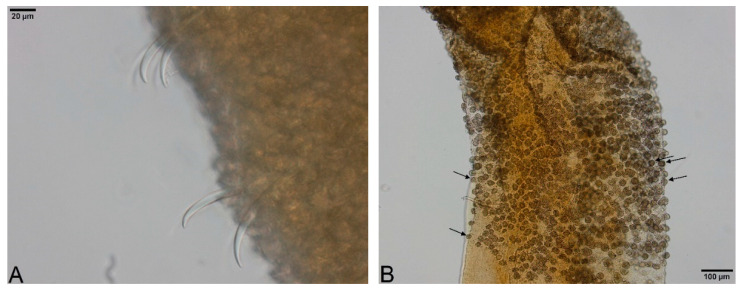
(**A**): Ventral chaetae of *Embolocephalus velutinus*. (**B**): Large dark and roundish particle aggregates of *Spirosperma ferox* (here, middle part). Author: Régis Vivien.

**Figure 5:**
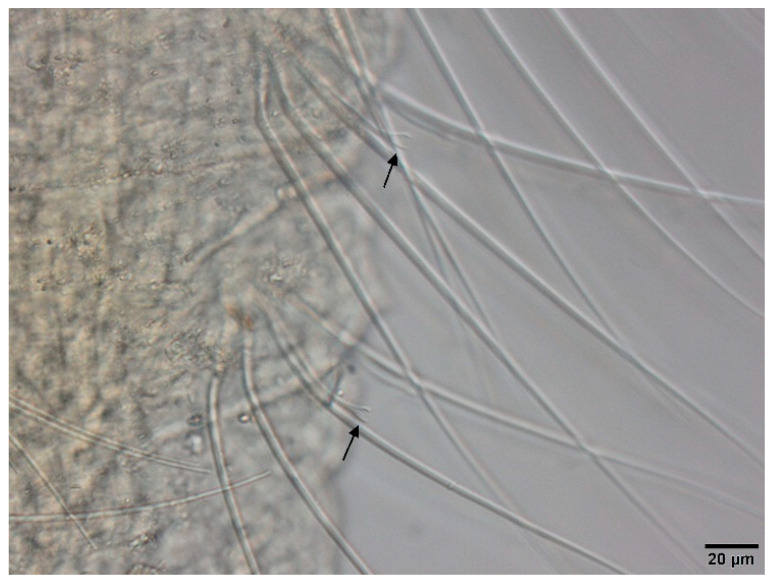
Anterior dorsal chaetae of *Spirosperma ferox*. Author: Régis Vivien.

**Figure 6:**
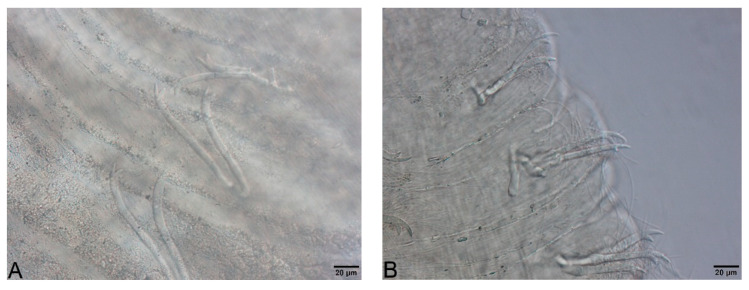
(**A**): Anterior ventral chaetae (segments II to IV) of *Spirosperma ferox*. (**B**): Anterior ventral chaetae (segments II to IV) of *Quistadrilus multisetosus*. Author: Régis Vivien.

**Figure 7:**
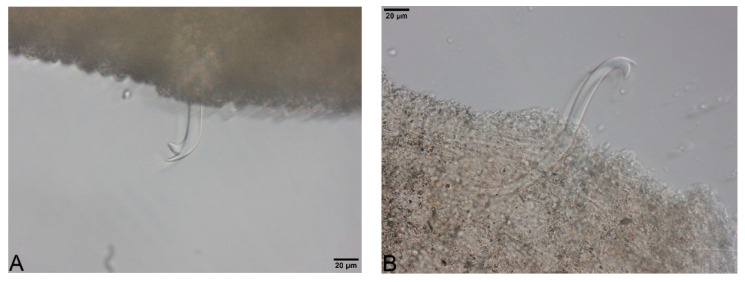
(**A**): Posterior ventral chaeta of *Spirosperma ferox*. (**B**): Posterior ventral chaeta of *Quistadrilus multisetosus*. Author: Régis Vivien.

1. Large, dark and roundish particle aggregates arranged randomly, covering the whole body surface, often hiding the chaetae (Figure 3A); Irregular and small dark particle aggregates arranged in transversal lines in some parts of the body surface, but almost always completely hidden by the large dark particle aggregates; In ventral bundles, simple-pointed and finely bifid chaetae (Figure 4A); In anterior dorsal bundles, chaetae are bifid with short inconspicuous teeth, these chaetae are mostly hidden by the large dark particle aggregates; Presence of prominent light sensory papillae arranged in a transversal row in each segment on the chaetal line but hidden by the large dark particle aggregates; Prostomium not elongated (Figure 3A) *Embolocephalus velutinus* *
  Shape of the chaetae different, all the ventral chaetae bifid 2
2. Large, dark and roundish particle aggregates similar to those of *E. velutinus*, arranged randomly, covering a large part of the body surface (Figure 4B); Irregular and small dark particle aggregates arranged in transversal lines in some parts of the body surface, often also present in the anterior part (Figure 3B); Absence of prominent light sensory papillae; Prostomium not or slightly elongated (Figure 3B); In anterior dorsal bundles, pectinate lyre-shaped chaetae with short teeth (Figure 5); In anterior ventral bundles, chaetae are bifid with upper tooth as long or 1.5-fold longer than the lower one (Figure 6A); In posterior ventral bundles, chaetae are bifid with a large lower tooth and a thin upper tooth (Figure 7A); Posterior ventral chaetae sometimes absent or inconspicuous (hidden by the large dark particle aggregates) in some segments *Spirosperma ferox* *

**Figure 8:**
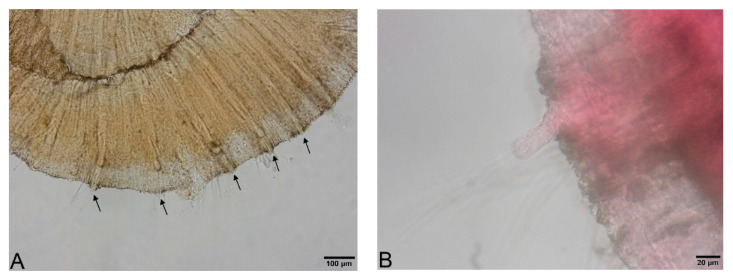
(**A**): Segment of *Quistadrilus multisetosus* with some prominent light sensory papillae arranged in transversal rows on the chaetal line. (**B**): Detail of a prominent light sensory papilla of *Quistadrilus multisetosus*. Author: Régis Vivien.

**Figure 9:**
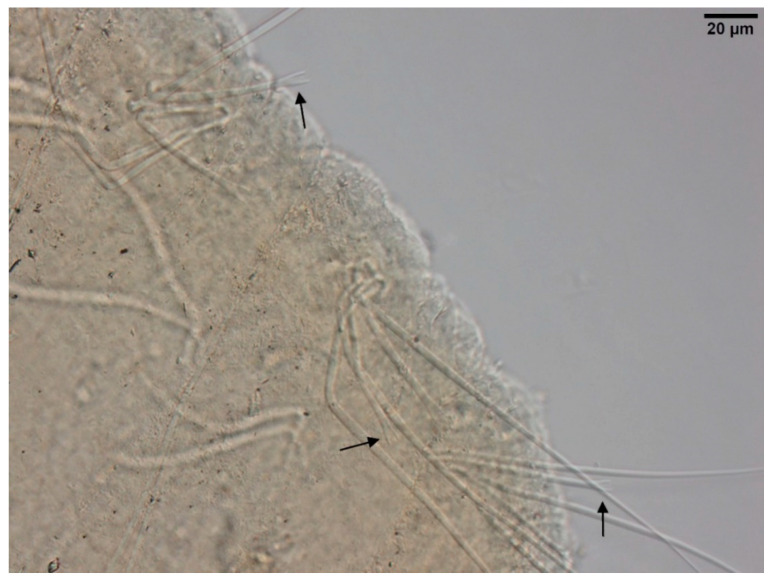
Anterior dorsal chaetae of *Quistadrilus multisetosus* (segments II–IV). Author: Régis Vivien.

Irregular and small dark particle aggregates arranged in transversal lines in some parts of the body surface (Figure 3C); Sometimes, presence of large, dark and roundish particle aggregates on the body surface, but few and localized; Presence of prominent light sensory papillae arranged in a transversal row in each segment on the chaetal line (Figure 8A,B) but often not well visible on fixed specimens; Prostomium elongated (Figure 3C); In anterior dorsal bundles, pectinate chaetae with long and straight teeth (Figure 9); In anterior ventral bundles, chaetae are bifid with upper tooth generally 1.5 to 2.5 fold longer than the lower tooth (Figure 6B); In posterior ventral bundles, chaetae are bifid and strongly sigmoid, with a large and curved lower tooth and a thinner and shorter upper tooth (Figure 7B); Posterior ventral chaetae always present and well visible in each segment *Quistadrilus multisetosus*
  * one specimen of *S. ferox* and one specimen of *E. velutinus* without any large dark and roundish particle aggregates were found in Lake Geneva; the specimen of *E. velutinus* presented small dark particle aggregates arranged in transversal lines in some parts of the body surface.

Table 2 summarizes the morphological features allowing distinction between *Q. multisetosus* and *S. ferox*.

### 3.3. Examination of Specimens from Collections

We examined ten specimens identified as *Spirosperma ferox* collected at four different sites of the Vidy Bay (in 1974), located downstream of the WWTP of the city of Lausanne at different distances from the outlet. All specimens belonged to *Quistadrilus multisetosus* according to the above-mentioned morphological characters, which demonstrates that this species was already present in the Vidy Bay in 1974. In Supplementary Figures S1–S12, photos of three of these specimens are provided. For each specimen (No1-3), some features allowing to identify *Q. multisetosus* (elongated prostomium, absence of large dark and roundish particle aggregates, presence of fine dark particle aggregates, shape of the anterior ventral and dorsal chaetae and of the posterior ventral chaetae) are shown. The prominent light sensory papillae are not or not well visible on these specimens (therefore not shown).

### 3.4. Phylogenetic Analysis

**Figure 10:**
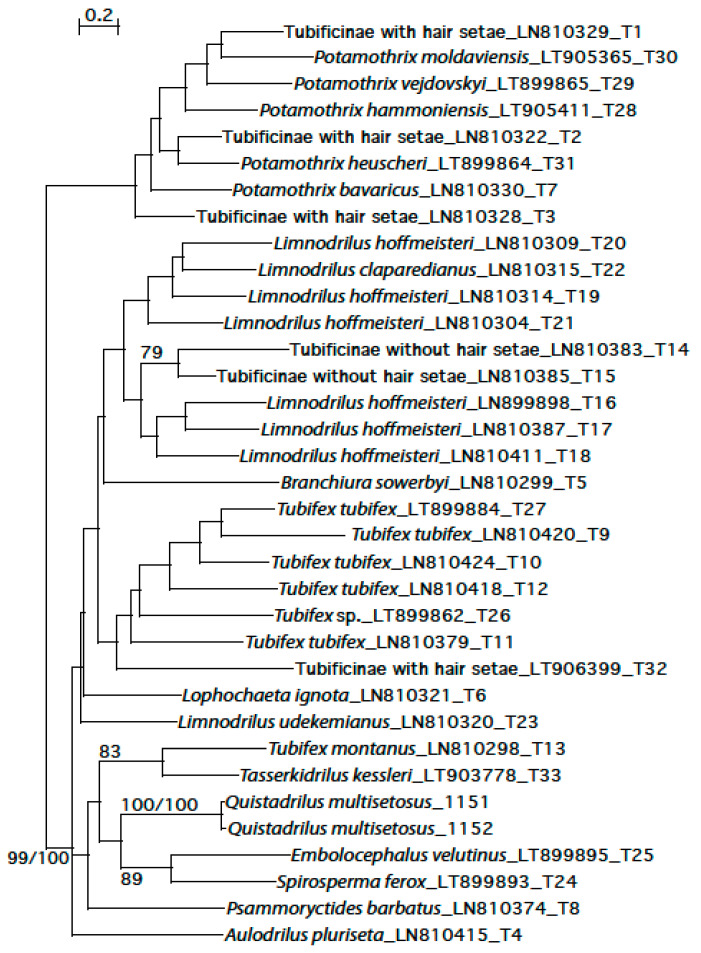
PhyML tree based on COI barcoding fragment of 35 sequences showing the position of *Quistadrilus multisetosus* within the Tubificinae. All lineages are separated by ≥10% of genetic divergence. The numbers placed at the internal nodes correspond to bootstrap values of ML and FastMe distance analyses; only those higher than 70% are indicated. For each lineage, the name of the taxon is indicated, followed by GenBank accession number and lineage name (of our Swiss database) or by the respective isolate numbers (for *Q. multisetosus*).

The obtained tree (Figure 10) is divided into four clades. A first clade including members of the genus *Potamothrix* and the Tubificinae sp. T1-3 (that probably belong to *Potamothrix*) branches at the base of the other clades. This is the only clade whose branching is supported (BV of 99 and 100%). A second clade consists of *Aulodrilus pluriseta* (Piguet, 1906) and *Psammoryctides barbatus* (Grube, 1861), the latter branching at the base of two sister clades containing *Tubifex montanus* Kowalewski, 1919 and *Tasserkidrilus kessleri* (Hrabe, 1962) (83%BV), and *Embolocephalus velutinus* and *Spirosperma ferox* (89% BV) with *Quistadrilus multisetosus* at their base. A third clade contains *Limnodrilus udekemianus* Claparede, 1862 and *Lophochaeta ignota* (Stolc, 1886) branching at the base of *Tubifex* spp. and Tubificinae sp. T32 (probably belonging to the genus *Tubifex*). The fourth clade consists of *Limnodrilus* spp. and two lineages of Tubificinae sp. (T14-15) (79%BV), probably belonging to the genus *Limnodrilus*, with *Branchiura sowerbyi* Beddard, 1892 branching at the base. The lineage of *Q. multisetosus* was separated from *S. ferox*, *E. velutinus*, *T. montanus* and *T. kessleri* by more than 20% of genetic variation (in COI). The maximum intra-lineage genetic divergence (in COI) of *Q. multisetosus* was 1.2%.

## 4. Discussion

*Quistadrilus multisetosus* is present at all investigated sites of the Vidy Bay, except sites 2 and 15, which are the farthest from the WWTP and CSO effluents. The species was found in low abundance on the shore at site 90 near the Vidy Bay and absent from all the other investigated sites. Its presence at site 90 seems to be explained only by the short distance between this site and the Vidy Bay that clearly constitutes a reservoir for this species in the lake. Genetic analyses confirmed the absence of the species at 8 sites, among them one (Site 32) sampled at two different times, and confirmed the high abundance of *Q. mutisetosus* at one site (53) in the Vidy Bay.

Considering the size of Lake Geneva, we investigated a relatively low number of sites and the number of specimens examined per site does not exceed 100 for half of the sampling sites. However, we selected the sites all around the lake and in particular on the shores where the probability to find *Quistadrilus multisetosus* was assumed to be the highest. This species has indeed only been found in two other Swiss lakes along shores ([9] and unpublished data). Given the low number of specimens examined per site, we considered it important to carry out an environmental DNA survey for some selected sites in order to confirm the results obtained by morphological analysis. 

Our study shows that *Quistadrilus multisetosus* tolerates strong organic matter pollution as it was found in high abundance under the influence of the effluents of a WWTP and a CSO. These results confirm the observations of Howmiller and Scott [43] and Vetricek and Sporka [5], who also detected *Q. multisetosus* in environments highly enriched with organic matter. As sediments in the Vidy Bay also contain high concentrations of metals, PCBs and PAHs, we can also suspect a high tolerance of *Q. multisetosus* to these contaminants. At sites 3 to 5 and 53, more than 90% of specimens belonged to resistant taxa to organic matter enrichment, according to the classification of oligochaetes in lakes by Lafont et al. [44]. The dominant species associated with *Q. multisetosus* were *Limnodrilus hoffmeisteri*, *Tubifex tubifex* (Muller, 1774), *Aulodrilus pluriseta*, *Potamothrix hammoniensis* (Michaelsen, 1901) and *Potamothrix vejdovskyi* (Hrabe, 1941) (Supplementary Table S1). On the other hand, at the two most distant sites from the WWTP and CSO in the Vidy Bay (sites 2 and 15), the structure of oligochaete communities indicated that sediments were well oxygenated as taxa sensitive to organic pollution (Lumbriculidae spp., *Stylodrilus heringianus* Claparede, 1862 and *Embolocephalus velutinus*, cf. [44]) were present in high abundance (44% and 50%, respectively) (Supplementary Table S1). The good biological quality observed at sites 2 and 15 could be explained by a reduction of the input of organic matter due to the distance, by unfavorable conditions for organic sedimentations such as strong currents and the steep bottom slope and/or by the presence of exfiltrations of groundwater (observed in some locations in Lake Geneva, [45]). At these two sites, the environmental conditions seemed unfavorable for the colonization of *Q. multisetosus*, and we can hypothesize that this species is competitive only in organically enriched sediments with a low level of oxygenation.

The capacity of *Quistadrilus multisetosus* to expand in Lake Geneva seems limited. According to our results, this species was already present in the Vidy Bay in 1974 and have not expanded since then. The reduction of phosphorus concentrations in water of this lake since the 1980s [46] has certainly not favored its dissemination. Some other introduced oligochaete species such as *Potamothrix vejdovskyi*, *Potamothrix hammoniensis*, *Potamothrix heuscheri* (Bretscher, 1900), *Potamothrix moldaviensis* Vejdovsky and Mrazek, 1903 and *Psammoryctides barbatus* have more successfully colonized Lake Geneva. Indeed, their presence has been reported at many locations in this lake. However, two foreign oligochaete species, *Psammoryctides moravicus* (Hrabe, 1934) and *Potamothrix bedoti* (Piguet, 1913) are known from only one (*P. moravicus*) or a few locations (*P. bedoti*) in Lake Geneva. The presence of *P. moravicus* was reported in 2018 [47] and it might have been recently introduced. *P. bedoti* was first reported in this lake in the 1960s [45], and this species could also have a limited capacity to expand in the lake. However, since this species can be identified (using morphology) only when specimens are mature and can reproduce by fragmentation [12], it is possible that its frequency in this lake is underestimated.

How and when *Quistadrilus multisetosus* was introduced in Lake Geneva is unknown. But the effluents of the WWTP of the city of Lausanne and of the CSO seem to be the source of this introduction. A plausible hypothesis is that this species was used (associated to other worms) in fishkeeping activity and released by the discharges of the WWTP and CSO. Worms sold for decades in aquarium shops as “Tubifex” are collected in polluted fine sediments [48] and can therefore include different species. *Q. multisetosus*, which is highly tolerant to pollution, could have thus been associated with other resistant Tubificinae such as *Tubifex tubifex* or *Limnodrilus hoffmeisteri* as food for aquarium fishes.

*Quistadrilus multisetosus* can be confounded with *Spirosperma ferox*, as they are morphologically similar. Our phylogenetic analysis confirms that the two species are clearly separated. The identification of *Q. multisetosus* specimens collected in Lake Geneva in 1974 as *S. ferox* is understandable given the resemblance of these two species and the absence of *Q. multisetosus* description in the identification keys of aquatic oligochaetes potentially present in Europe at that time. This misidentification led Lang and Lang-Dobler [49] to consider *S. ferox* as highly tolerant to organic matter pollution, even if this species had been described by several authors as sensitive to eutrophication. The identification key provided in the present work was conceived to easily differentiate *Q. multisetosus* from *S. ferox* and *Embolocephalus velutinus*. It includes several newly observed differential characters between these species, such as the shape of chaetae and prostomium. It could help to improve the monitoring of *Q. multisetosus* in aquatic ecosystems.

It is important to carry on the monitoring of *Quistadrilus multisetosus* in Lake Geneva, even if at present it does not seem to disseminate. The current oligo-mesotrophic conditions in Lake Geneva are certainly an unfavorable factor for a widespread colonization of *Q. multisetosus*. However, our knowledge of other environmental factors that influence this species is limited. In particular, the warming of waters, which tends to undermine the positive effects of reduction of eutrophication in lakes [50], might contribute to its expansion.

## Figures and Tables

**Table 1:** Details of the sampling and number of specimens identified per site. The Swiss coordinate system (1903) was used.

Site	Location	Date	Coordinates		Depth (m)	No Subsamples	No Specimens Identified
			x	y			
38	St Prex	20 April 2015	2,526,000	1,149,000	21	5	467
32	Buchillon	20 April 2015	2,522,000	1,146,600	22	5	220
30		20 April 2015	2,519,295	1,139,643	151	5	446
35		20 April 2015	2,523,230	1,144,720	149	5	295
49		20 April 2015	2,534,000	1,144,000	309	5	107
58		20 April 2015	2,539,000	1,145,000	309	5	162
32	Buchillon	26 October 2017	2,521,999	1,146,600	20–25	3	100
53	baie de Vidy	26 October 2017	2,534,721	1,151,336	42–44	3	100
78	Grangettes	26 October 2017	2,558,140	1,139,994	70	3	100
1	Vengeron	04 June 17	2,501,201	1,122,347	10	3	100
6	Mies	22 May 2018	2,503,999	1,127,985	54	3	100
21	Yvoire	22 May 2018	2,514,799	1,137,100	52	3	100
36	Thonon	22 May 2018	2,524,002	1,135,995	32	3	100
4	baie de Vidy	18 October 2016	2,535,725	1,151,289	24	3	100
3	baie de Vidy	17 October 2016	2,535,718	1,151,070	46	3	100
5	baie de Vidy	18 October 2016	2,535,699	1,150,839	60	3	100
2	baie de Vidy	18 October 2016	2,535,684	1,150,606	76	3	100
15	baie de Vidy	20 October 2016	2,535,592	1,149,562	188	3	100
90	St Sulpice	15 August 2019	2,531,708	1,150,583	14	3	100
91	Cully	28 August 2019	2,545,282	1,148,504	15	3	100
92	Chevrens	2009	2,506,000	1,128,000	70	3	235
93	Coppet	2009	2,504,340	1,129,700	20–22	3	418
94		2009	2,504,885	1,129,170	40	3	358
95	Tougues	2009	2,507,500	1,131,500	70	3	228
96	Founex	2009	2,505,360	1,132,000	20	3	281
97		2009	2,505,685	1,131,940	40	3	232
98	Nernier	2009	2,510,800	1,136,350	70	5	209
99	Nyon	2009	2,508,200	1,137,000	20	5	341
100		2009	2,508,605	1,136,880	40	5	299

**Table 2:** Summary of the morphological differences between *Quistadrilus multisetosus* and *Spirosperma ferox*, mostly based on the authors’ own observations.

Morphological Characters	*Spirosperma ferox*	*Quistadrilus multisetosus*
Prominent light sensory papillae	Absent	Present but often not well visible on fixed specimens
Large dark and roundish particle aggregates on the body surface	Present and abundant on all or a large part of the body *	Absent or few and localized
Small dark particle aggregates arranged in transversal lines on the body surface	Present, often hidden by the large dark particle aggregates	Present and generally conspicuous
Prostomium	Flattened, rarely slightly elongated	Always elongated
Anterior dorsal chaetae	Lyre-shaped and short teeth	Long and straight teeth
Anterior ventral chaetae	Upper tooth as long or 1.5-fold longer than the lower one	Upper tooth generally 1.5 to 2.5-fold longer than the lower one
Posterior ventral chaetae	Not strongly sigmoid; Lower tooth not or slightly curved and upper tooth as long or slightly shorter;	Strongly sigmoid; Curved lower tooth and shorter upper tooth;
	Sometimes absent or hidden by the large dark particle aggregates in some segments	Always well visible in each segment

* one specimen of *S. ferox* without any large dark and roundish particle aggregates was found in Lake Geneva.

## Supplementary Materials

The following are available online at https://www.mdpi.com/2079-7737/9/12/436/s1, Table S1: Faunistic data obtained with morphological analysis (sampling from 2016 to 2019): number of specimens of each taxon per site; Table S2: Faunistic data obtained with morphological analysis (sampling in 2009 and 2015): number of specimens of each taxon per site; Table S3: Faunistic data obtained with high-throughput sequencing: number of reads of each taxon, total number of reads (Total reads), total number of reads corresponding to oligochaetes (Total reads oligochaetes) and percentage of reads corresponding to oligochaetes (% reads oligochaetes) per sample; Figure S1: anterior part (elongated prostomium, absence of large dark roundish particle aggregates, presence of fine dark particle aggregates) of specimen No1 of *Quistadrilus multisetosus* collected in the Vidy Bay in 1974. Author: Régis Vivien; Figure S2: anterior ventral chaetae of specimen No1 of *Quistadrilus multisetosus* collected in the Vidy Bay in 1974. Author: Régis Vivien; Figure S3: anterior dorsal chaetae of specimen No1 of *Quistadrilus multisetosus* collected in the Vidy Bay in 1974. Author: Régis Vivien; Figure S4: posterior ventral chaetae of specimen No1 of *Quistadrilus multisetosus* collected in the Vidy Bay in 1974. Author: Régis Vivien; Figure S5: anterior part (elongated prostomium, absence of large dark roundish particle aggregates, presence of fine dark particle aggregates) of specimen No2 of *Quistadrilus multisetosus* collected in the Vidy Bay in 1974. Author: Régis Vivien; Figure S6: anterior ventral chaetae of specimen No2 of *Quistadrilus multisetosus* collected in the Vidy Bay in 1974. Author: Régis Vivien; Figure S7: anterior dorsal chaetae of specimen No2 of *Quistadrilus multisetosus* collected in the Vidy Bay in 1974. Author: Régis Vivien; Figure S8: posterior ventral chaetae of specimen No2 of *Quistadrilus multisetosus* collected in the Vidy Bay in 1974. Author: Régis Vivien; Figure S9: anterior part (elongated prostomium, absence of large dark roundish particle aggregates, presence of fine dark particle aggregates) of specimen No3 of *Quistadrilus multisetosus* collected in the Vidy Bay in 1974. Author: Régis Vivien; Figure S10: anterior ventral chaetae of specimen No3 of *Quistadrilus multisetosus* collected in the Vidy Bay in 1974. Author: Régis Vivien; Figure S11: anterior dorsal chaetae of specimen No3 of *Quistadrilus multisetosus* collected in the Vidy Bay in 1974. Author: Régis Vivien; Figure S12: posterior ventral chaetae of specimen No3 of *Quistadrilus multisetosus* collected in the Vidy Bay in 1974. Author: Régis Vivien.
