# Supplementary material for: The Foreign Oligochaete Species Quistadrilus multisetosus (Smith, 1900) in Lake Geneva: Morphological and Molecular Characterization and Environmental Influences on Its Distribution
Source: Biology (Basel). 2020 Dec 1;9(12):436. doi: 10.3390/biology9120436 (PMC7760516; doi:10.3390/biology9120436)
Supplement: Supplementary file 1 [file biology-09-00436-s001.zip › Supplementary_Table_S1.docx]

| **Site** | **1** | **32** | **53** | **78** | **6** | **36** | **21** | **4** | **3** | **5** | **2** | **15** | **91** | **90** |
| --- | --- | --- | --- | --- | --- | --- | --- | --- | --- | --- | --- | --- | --- | --- |
| **Sampling year** | **2017** | **2017** | **2017** | **2017** | **2018** | **2018** | **2018** | **2016** | **2016** | **2016** | **2016** | **2016** | **2019** | **2019** |
| Tubificinae with hair setae (unidentifiable) | 33 | 18 | 50 | 17 | 36 | 18 | 6 | 48 | 43 | 36 | 10 | 11 | 22 | 12 |
| *Tubifex tubifex* |  |  | 6 | 1 | 2 | 4 |  | 1 |  | 5 | 3 | 2 |  |  |
| *Aulodrilus pluriseta* |  | 12 |  | 2 | 1 | 2 |  | 2 |  | 3 | 2 |  | 4 | 1 |
| *Psammoryctides barbatus* | 2 |  |  | 16 | 10 | 2 |  |  | 4 |  |  |  |  | 1 |
| *Psammoryctides moravicus* | 3 |  |  |  |  |  |  |  |  |  |  |  |  |  |
| *Embolocephalus velutinus* |  |  |  |  |  | 18 | 24 |  |  |  | 23 | 1 |  | 1 |
| *Spirosperma ferox* | 5 |  |  |  | 6 | 6 | 4 |  |  |  |  |  | 1 |  |
| *Potamothrix heuscheri* | 1 | 1 |  | 1 | 3 |  |  |  |  | 1 |  | 1 | 1 | 1 |
| *Potamothrix hammoniensis* |  |  |  | 1 | 3 |  |  | 1 | 2 | 6 | 3 | 2 |  |  |
| *Potamothrix vejdovskyi* | 1 | 5 |  | 29 |  | 2 | 1 | 1 |  | 13 | 3 | 4 | 15 | 11 |
| *Quistadrilus multisetosus* |  |  | 13 |  |  |  |  | 8 | 32 | 12 |  |  |  | 2 |
| *Potamothrix bavaricus* |  |  |  |  |  |  |  |  | 1 |  |  |  |  |  |
| *Potamothrix bedoti* |  |  |  |  |  |  |  |  |  |  |  |  | 1 |  |
| *Lophochaeta ignota* |  |  |  |  |  |  |  |  | 1 | 1 |  |  |  | 2 |
| Tubificinae without hair setae (unidentifiable) | 25 | 39 | 22 | 18 | 29 | 28 | 25 | 35 | 12 | 13 | 19 | 20 | 30 | 54 |
| *Limnodrilus hoffmeisteri* | 11 | 2 | 11 | 2 | 4 | 1 | 3 | 3 |  |  | 15 | 7 | 4 | 3 |
| *Limnodrilus claparedianus* |  |  |  | 1 | 1 | 2 |  |  |  | 1 |  |  |  | 2 |
| *Limnodrilus profundicola* |  | 1 | 1 | 2 | 3 |  | 1 |  |  |  |  |  | 1 |  |
| *Limnodrilus udekemianus* |  |  |  |  |  |  |  |  |  |  |  |  | 1 |  |
| *Aulodrilus limnobius* | 3 | 6 |  |  |  |  |  |  |  |  |  |  |  | 1 |
| *Potamothrix moldaviensis* | 7 |  |  |  |  | 6 | 5 |  |  | 1 | 1 | 3 | 3 | 4 |
| Lumbriculidae (unidentifiable) | 1 |  |  |  | 4 | 10 | 3 |  |  | 1 | 9 | 26 | 7 |  |
| *Stylodrilus heringianus* |  |  |  |  |  |  |  |  |  |  | 11 | 18 | 1 |  |
| *Stylodrilus lemani* |  |  |  |  | 1 |  |  |  |  |  |  | 4 |  |  |
| *Lumbriculus variegatus* | 2 |  |  | 1 |  |  |  |  |  | 2 |  |  |  |  |
| *Ophidonais serpentina* |  |  |  |  |  |  |  |  |  | 1 |  |  |  |  |
| *Piguetiella blanci* | 5 | 3 |  | 1 |  | 2 |  |  |  | 1 |  |  | 1 |  |
| *Specaria josinae* |  |  |  |  |  |  |  |  | 1 |  |  |  |  |  |
| *Uncinais uncinata* |  |  |  |  |  |  |  |  | 1 | 3 | 1 |  | 6 |  |
| *Vejdovskyella intermedia* |  |  |  | 6 |  |  |  |  | 2 |  |  | 1 | 1 | 1 |
| *Stylaria lacustris* |  | 10 |  |  |  | 2 |  |  |  |  |  |  | 1 | 2 |
| *Nais pardalis* |  |  |  |  |  |  |  |  | 1 |  |  |  |  |  |
| *Dero digitata* | 1 | 2 |  |  |  |  |  | 1 |  |  |  |  |  |  |
| *Chaetogaster diaphanus* |  |  |  |  |  |  |  |  |  |  |  |  |  | 2 |

Supplementary table S1: Faunistic data obtained with morphological analysis (sampling from 2016 to 2019): number of specimens of each taxon per site
