# Supplementary material for: The Foreign Oligochaete Species Quistadrilus multisetosus (Smith, 1900) in Lake Geneva: Morphological and Molecular Characterization and Environmental Influences on Its Distribution
Source: Biology (Basel). 2020 Dec 1;9(12):436. doi: 10.3390/biology9120436 (PMC7760516; doi:10.3390/biology9120436)
Supplement: Supplementary file 1 [file biology-09-00436-s001.zip › Supplementary_Table_S2.docx]

| **Site** | **92** | **93** | **94** | **95** | **96** | **97** | **98** | **99** | **100** | **49** | **58** | **30** | **35** | **32** | **38** |
| --- | --- | --- | --- | --- | --- | --- | --- | --- | --- | --- | --- | --- | --- | --- | --- |
| **Sampling year** | **2009** | **2009** | **2009** | **2009** | **2009** | **2009** | **2009** | **2009** | **2009** | **2015** | **2015** | **2015** | **2015** | **2015** | **2015** |
| Tubificinae with hair setae (unidentifiable) | 130 | 234 | 195 | 79 | 125 | 109 | 66 | 99 | 182 | 69 | 130 | 242 | 115 | 121 | 52 |
| *Tubifex tubifex* | 3 | 5 | 0 | 2 | 0 | 1 | 2 | 1 | 0 | 13 | 0 | 3 | 1 | 0 | 0 |
| *Aulodrilus pluriseta* | 3 | 1 | 0 | 0 | 8 | 3 | 5 | 27 | 7 | 0 | 0 | 0 | 0 | 8 | 46 |
| *Psammoryctides barbatus* | 0 | 0 | 0 | 0 | 1 | 7 | 0 | 1 | 0 | 0 | 0 | 0 | 0 | 0 | 0 |
| *Embolocephalus velutinus* | 0 | 0 | 12 | 2 | 0 | 4 | 6 | 0 | 0 | 0 | 0 | 5 | 36 | 0 | 0 |
| *Spirosperma ferox* | 0 | 6 | 4 | 0 | 4 | 12 | 0 | 1 | 3 | 0 | 0 | 0 | 0 | 3 | 0 |
| *Potamothrix heuscheri* | 1 | 3 | 7 | 3 | 10 | 1 | 12 | 7 | 4 | 11 | 17 | 12 | 2 | 3 | 1 |
| *Potamothrix hammoniensis* | 4 | 33 | 3 | 0 | 18 | 2 | 0 | 5 | 3 | 0 | 7 | 0 | 0 | 0 | 1 |
| *Potamothrix vejdovskyi* | 44 | 1 | 20 | 53 | 1 | 5 | 16 | 35 | 3 | 0 | 0 | 44 | 0 | 25 | 10 |
| Tubificinae without hair setae (unidentifiable) | 45 | 77 | 84 | 81 | 70 | 69 | 87 | 105 | 60 | 0 | 0 | 57 | 67 | 42 | 22 |
| *Limnodrilus hoffmeisteri* | 3 | 30 | 15 | 4 | 33 | 18 | 6 | 34 | 36 | 1 | 0 | 1 | 0 | 6 | 8 |
| *Limnodrilus profundicola* | 0 | 0 | 0 | 0 | 0 | 0 | 0 | 0 | 0 | 0 | 0 | 3 | 2 | 0 | 0 |
| *Potamothrix moldaviensis* | 2 | 0 | 18 | 4 | 1 | 0 | 2 | 19 | 0 | 0 | 0 | 14 | 42 | 0 | 0 |
| Tubificinae (unidentifiable) | 0 | 0 | 0 | 0 | 0 | 0 | 0 | 0 | 0 | 13 | 8 | 0 | 0 | 0 | 320 |
| *Stylodrilus heringianus* | 0 | 0 | 0 | 0 | 1 | 0 | 2 | 0 | 0 | 0 | 0 | 60 | 21 | 0 | 0 |
| *Stylodrilus lemani* | 0 | 17 | 0 | 0 | 4 | 1 | 5 | 3 | 0 | 0 | 0 | 5 | 9 | 0 | 0 |
| *Ophidonais serpentina* | 0 | 8 | 0 | 0 | 1 | 0 | 0 | 0 | 0 | 0 | 0 | 0 | 0 | 0 | 0 |
| *Piguetiella blanci* | 0 | 0 | 0 | 0 | 0 | 0 | 0 | 0 | 0 | 0 | 0 | 0 | 0 | 9 | 2 |
| *Specaria josinae* | 0 | 1 | 0 | 0 | 2 | 0 | 0 | 0 | 0 | 0 | 0 | 0 | 0 | 0 | 0 |
| *Uncinais uncinata* | 0 | 2 | 0 | 0 | 0 | 0 | 0 | 0 | 1 | 0 | 0 | 0 | 0 | 0 | 5 |
| *Vejdovskyella intermedia* | 0 | 0 | 0 | 0 | 2 | 0 | 0 | 4 | 0 | 0 | 0 | 0 | 0 | 2 | 0 |
| *Nais simplex* | 0 | 0 | 0 | 0 | 0 | 0 | 0 | 0 | 0 | 0 | 0 | 0 | 0 | 1 | 0 |

Supplementary table S2: Faunistic data obtained with morphological analysis (sampling in 2009 and 2015): number of specimens of each taxon per site
