# Supplementary material for: The Foreign Oligochaete Species Quistadrilus multisetosus (Smith, 1900) in Lake Geneva: Morphological and Molecular Characterization and Environmental Influences on Its Distribution
Source: Biology (Basel). 2020 Dec 1;9(12):436. doi: 10.3390/biology9120436 (PMC7760516; doi:10.3390/biology9120436)
Supplement: Supplementary file 1 [file biology-09-00436-s001.zip › Supplementary_Table_S3.docx]

| Site | 1 | 32 | 53 | 78 | 6 | 21 | 36 | 32 | 35 | 38 |
| --- | --- | --- | --- | --- | --- | --- | --- | --- | --- | --- |
| Sampling date | 04.09.2017 | 26.10.2017 | 26.10.2017 | 26.10.2017 | 22.05.2018 | 22.05.2018 | 22.05.2018 | 20.04.2015 | 20.04.2015 | 20.04.2015 |
| LL3_918_Stylodrilus_heringianus | 0 | 0 | 0 | 0 | 0 | 0 | 2703 | 0 | 503 | 0 |
| Stylodrilus_lemani_O13 | 0 | 0 | 0 | 0 | 2 | 0 | 1987 | 0 | 2 | 0 |
| N1_LN810268_Chaetogaster_diaphanus | 0 | 0 | 37 | 0 | 0 | 0 | 0 | 0 | 0 | 0 |
| N12_984_Nais_stolci_pardalis | 0 | 0 | 0 | 0 | 0 | 166 | 0 | 0 | 0 | 0 |
| N14_1010_Uncinais_uncinata | 0 | 0 | 0 | 316 | 0 | 0 | 146 | 0 | 0 | 0 |
| N5_966_Ophidonais_serpentina | 0 | 0 | 2 | 0 | 0 | 0 | 0 | 0 | 0 | 0 |
| N6_LN810254_Piguetiella_blanci | 855 | 266 | 0 | 0 | 0 | 0 | 0 | 0 | 0 | 0 |
| N7_820_Vejdovskyella_intermedia | 0 | 0 | 0 | 314 | 0 | 35 | 98 | 6 | 0 | 19 |
| Stylaria_lacustris_F1 | 0 | 0 | 0 | 37 | 0 | 0 | 33 | 0 | 0 | 0 |
| Quistadrilus_multisetosus_1149 | 0 | 0 | 9987 | 0 | 0 | 0 | 0 | 0 | 0 | 0 |
| T11_773_Tubifex_tubifex | 0 | 0 | 4043 | 71 | 207 | 0 | 0 | 0 | 168 | 0 |
| T12_986_Tubifex_tubifex | 0 | 0 | 390 | 0 | 2 | 0 | 0 | 0 | 0 | 0 |
| T15_LN810385_Tubificinae_sp | 9736 | 1592 | 19 | 0 | 0 | 0 | 0 | 0 | 0 | 7489 |
| Aulodrilus_limnobius_F34 | 6506 | 0 | 0 | 0 | 0 | 0 | 0 | 0 | 0 | 0 |
| Limnodrilus_claparedianus_cervix_L32 | 27 | 0 | 6 | 0 | 0 | 0 | 0 | 0 | 0 | 0 |
| Limnodrilus_profundicola_O3 | 0 | 0 | 1 | 0 | 4 | 0 | 0 | 0 | 0 | 0 |
| Limnodrilus_udekemianus_J9 | 0 | 0 | 3 | 0 | 0 | 0 | 0 | 0 | 0 | 0 |
| T17_755_Limnodrilus_hoffmeisteri | 0 | 150 | 11546 | 8 | 0 | 0 | 0 | 0 | 0 | 0 |
| T18_858_Limnodrilus_hoffmeisteri | 26 | 0 | 76 | 0 | 0 | 99 | 0 | 138 | 0 | 43 |
| T2_838_Tubificinae_sp | 754 | 0 | 2843 | 679 | 4 | 0 | 598 | 141 | 0 | 0 |
| T20_859_Limnodrilus_hoffmeisteri | 0 | 213 | 167 | 139 | 28 | 370 | 3 | 0 | 0 | 91 |
| T24_822_Spirosperma_ferox | 0 | 0 | 0 | 0 | 0 | 0 | 388 | 0 | 0 | 0 |
| T25_824_Embolocephalus_velutinus | 0 | 0 | 0 | 0 | 0 | 0 | 625 | 0 | 18 | 0 |
| T26_844_Tubifex_sp | 71 | 0 | 0 | 0 | 0 | 0 | 0 | 0 | 0 | 6468 |
| T27_856_Tubifex_tubifex | 0 | 0 | 344 | 0 | 0 | 0 | 0 | 0 | 0 | 0 |
| T28_834_Potamothrix_hammoniensis | 0 | 0 | 42 | 467 | 497 | 0 | 0 | 0 | 0 | 0 |
| T29_848_Potamothrix_vejdovskyi | 0 | 267 | 0 | 9978 | 0 | 0 | 0 | 439 | 0 | 71 |
| T3_LN810328_Tubificinae_sp | 0 | 0 | 0 | 0 | 0 | 0 | 0 | 0 | 0 | 75 |
| T30_752_Potamothrix_moldaviensis | 704 | 0 | 0 | 412 | 16 | 264 | 234 | 0 | 4524 | 0 |
| T31_847_Potamothrix_heuscheri | 165 | 118 | 399 | 374 | 2651 | 0 | 0 | 201 | 230 | 61 |
| T4_843_Aulodrilus_pluriseta | 0 | 2258 | 0 | 0 | 1287 | 0 | 0 | 0 | 0 | 0 |
| T5_LN810299_Branchiura_sowerbyi | 19 | 0 | 0 | 0 | 0 | 0 | 0 | 0 | 0 | 0 |
| T8_LN810374_Psammoryctides_barbatus | 0 | 0 | 0 | 57 | 8 | 0 | 2 | 0 | 0 | 0 |
| T9_LN810420_Tubifex_tubifex | 0 | 0 | 2118 | 0 | 1 | 0 | 0 | 0 | 0 | 0 |
| Tubificinae_sp_H27 | 0 | 0 | 0 | 103 | 0 | 0 | 0 | 0 | 0 | 0 |
| Total reads | 180024 | 215906 | 96680 | 171785 | 257318 | 118977 | 89757 | 66289 | 147509 | 146404 |
| Total reads oligochaetes | 18863 | 4864 | 32023 | 12955 | 4707 | 934 | 6817 | 925 | 5445 | 14317 |
| % reads oligochaetes | 10.5 | 2.3 | 33.1 | 7.5 | 1.8 | 0.8 | 7.6 | 1.4 | 3.7 | 9.8 |

Supplementary table S3: Faunistic data obtained with high-throughput sequencing: number of reads of each taxon, total number of reads (Total reads), total number of reads corresponding to oligochaetes (Total reads oligochaetes) and percentage of reads corresponding to oligochaetes (% reads oligochaetes) per sample
