# Supplementary figures and images for: The Foreign Oligochaete Species Quistadrilus multisetosus (Smith, 1900) in Lake Geneva: Morphological and Molecular Characterization and Environmental Influences on Its Distribution
Source: Biology (Basel). 2020 Dec 1;9(12):436. doi: 10.3390/biology9120436 (PMC7760516; doi:10.3390/biology9120436)

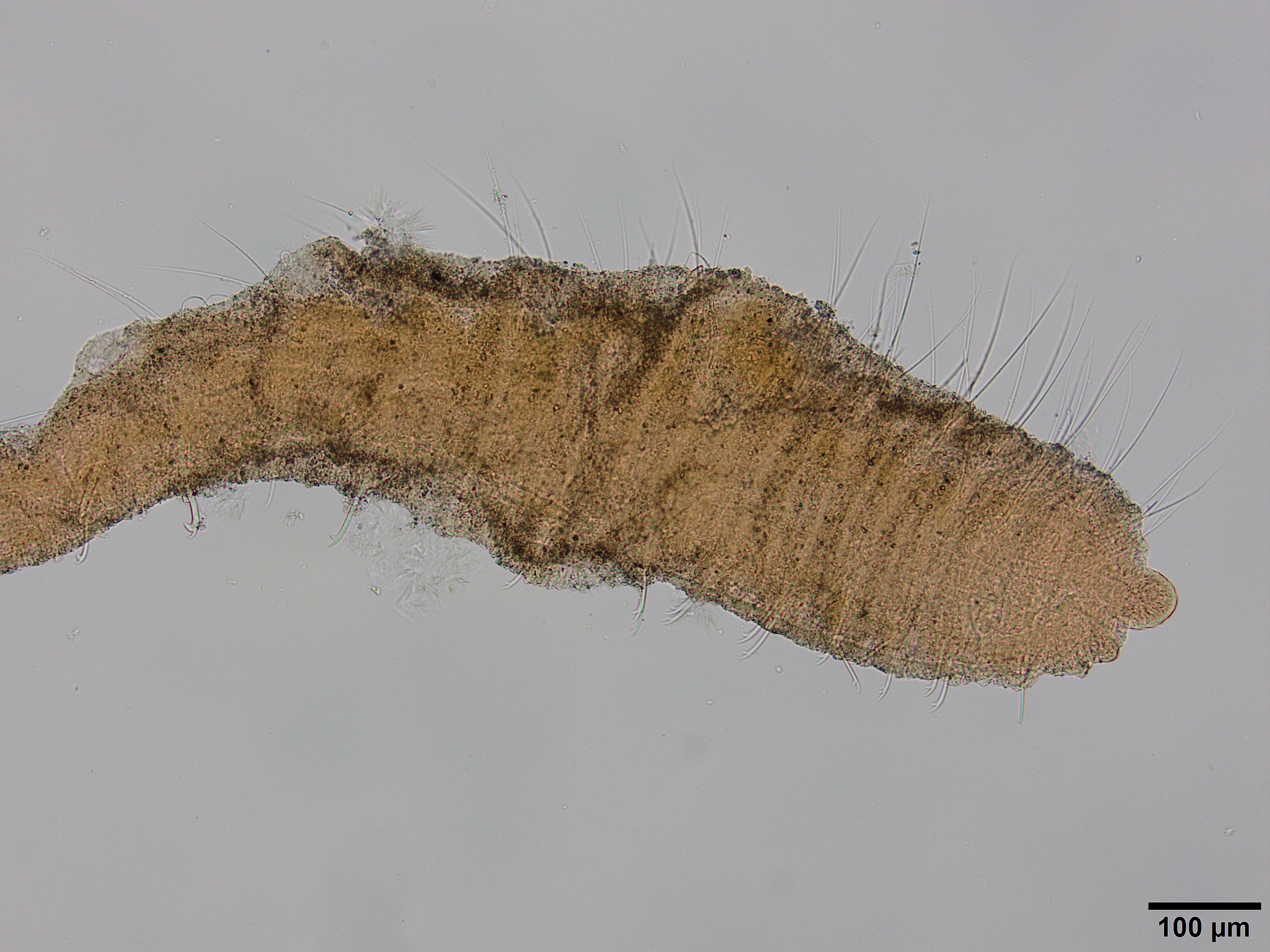

Supplement: Supplementary file 1 [file biology-09-00436-s001.zip › Supplementary_Figure_S1.jpg]

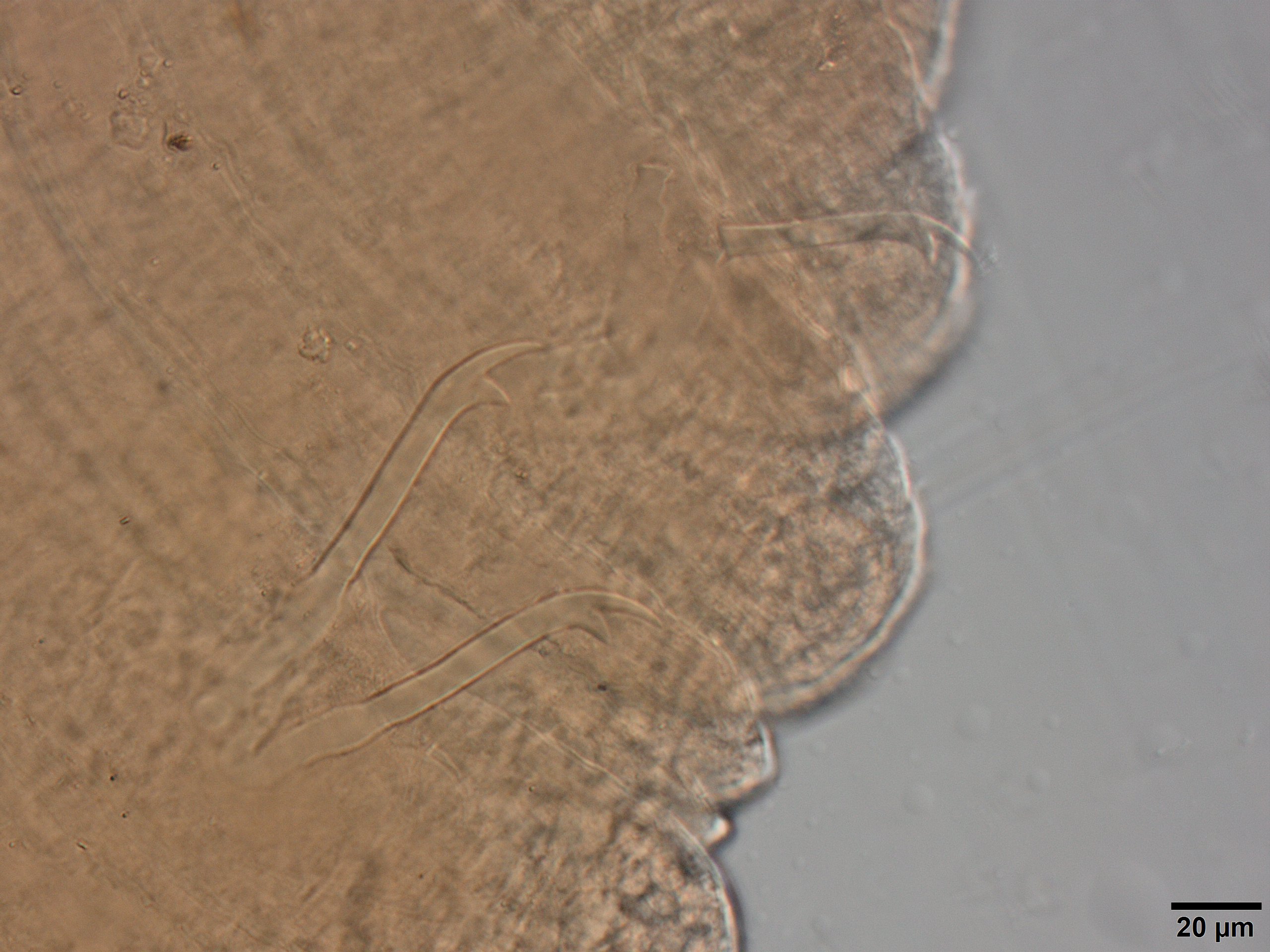

Supplement: Supplementary file 1 [file biology-09-00436-s001.zip › Supplementary_Figure_S10.jpg]

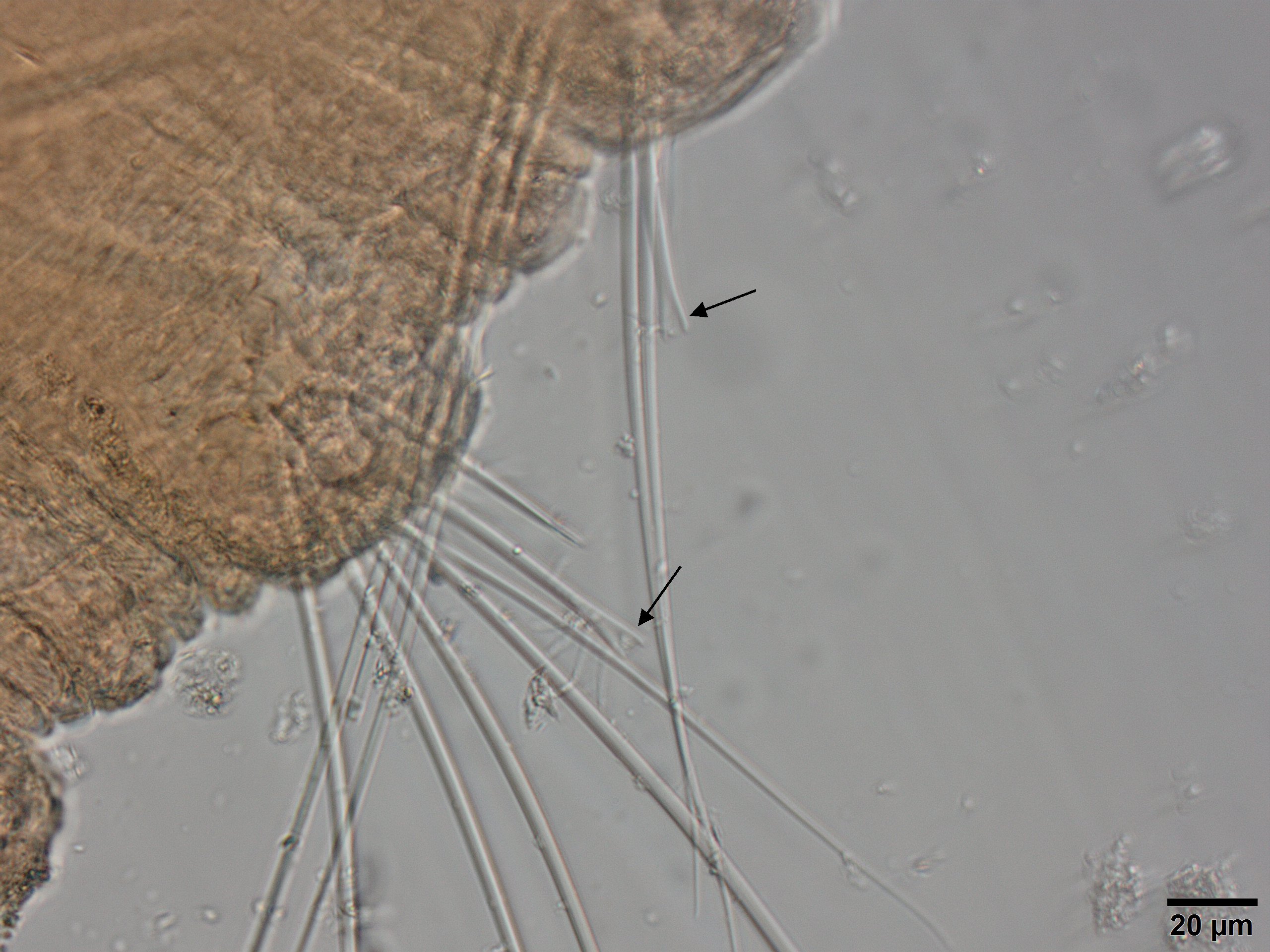

Supplement: Supplementary file 1 [file biology-09-00436-s001.zip › Supplementary_Figure_S11.jpg]

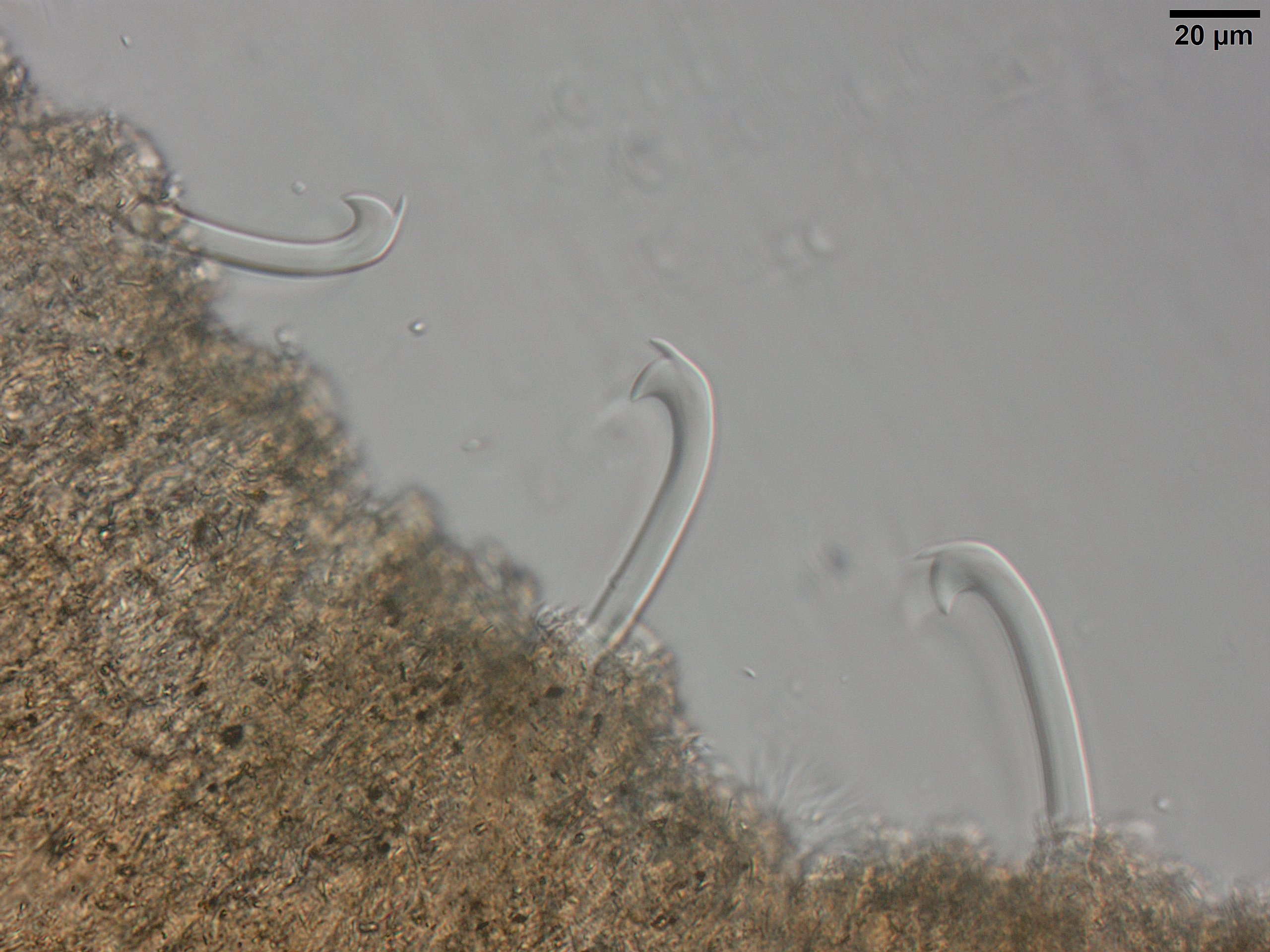

Supplement: Supplementary file 1 [file biology-09-00436-s001.zip › Supplementary_Figure_S12.jpg]

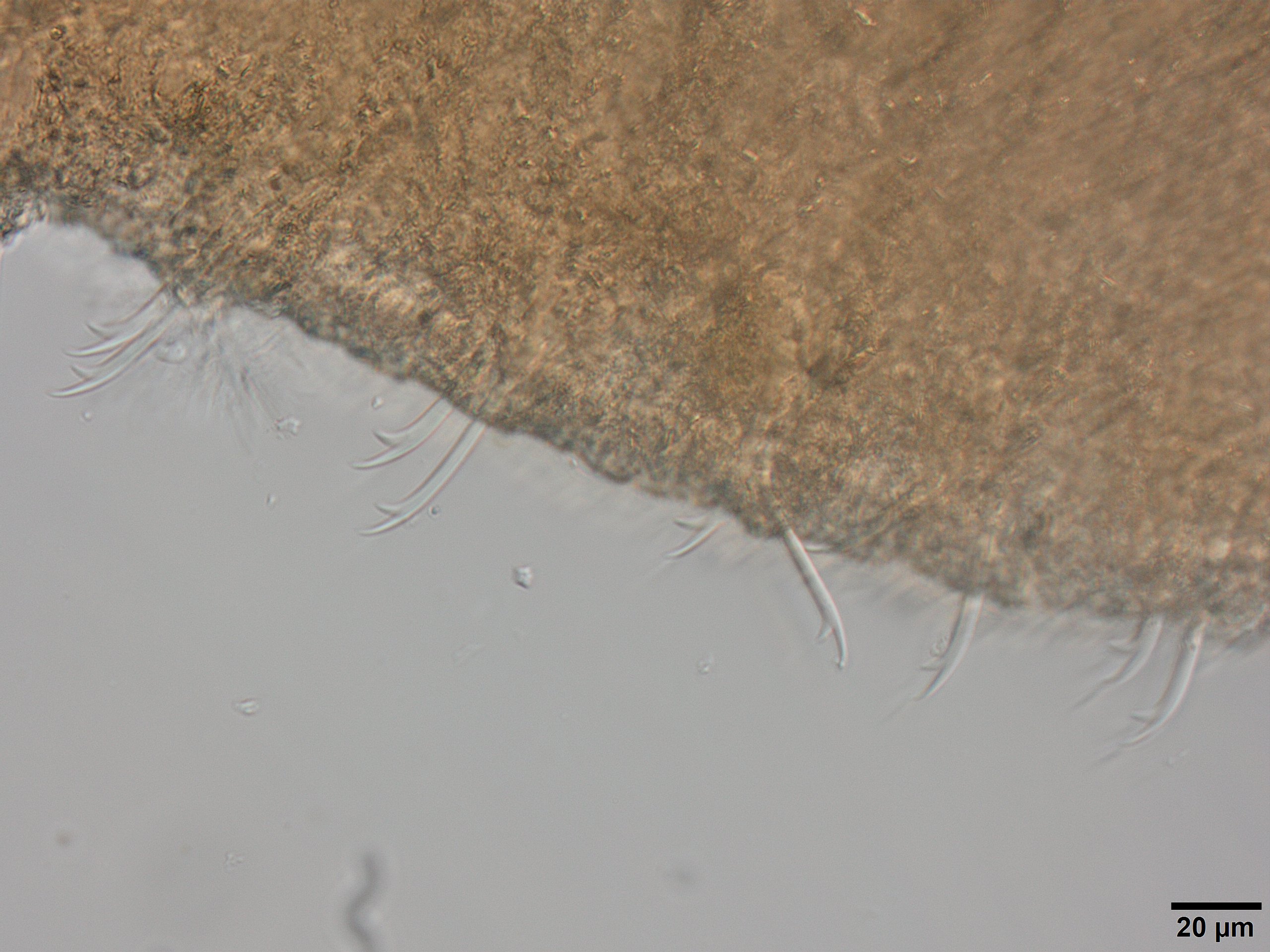

Supplement: Supplementary file 1 [file biology-09-00436-s001.zip › Supplementary_Figure_S2.jpg]

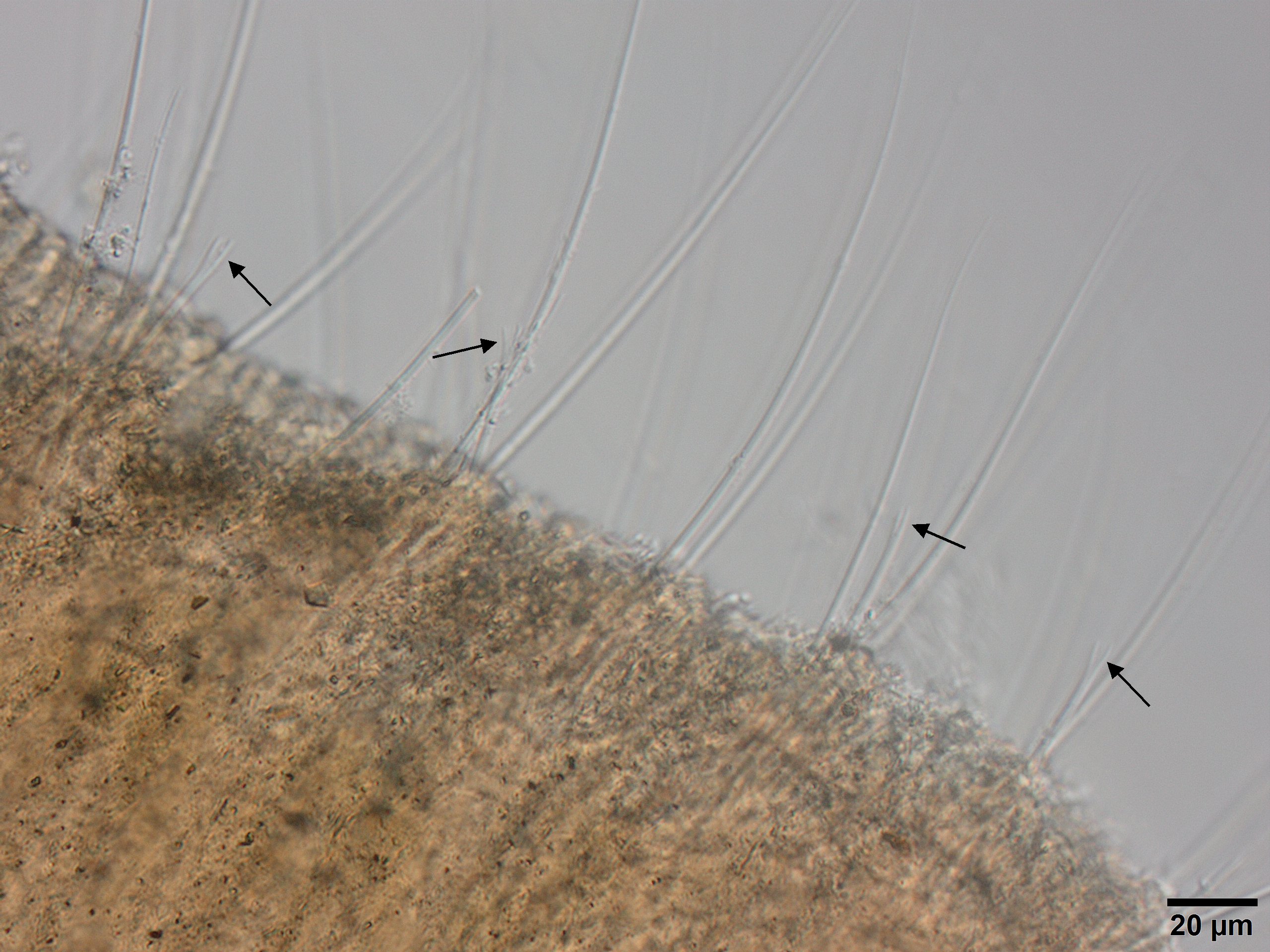

Supplement: Supplementary file 1 [file biology-09-00436-s001.zip › Supplementary_Figure_S3.jpg]

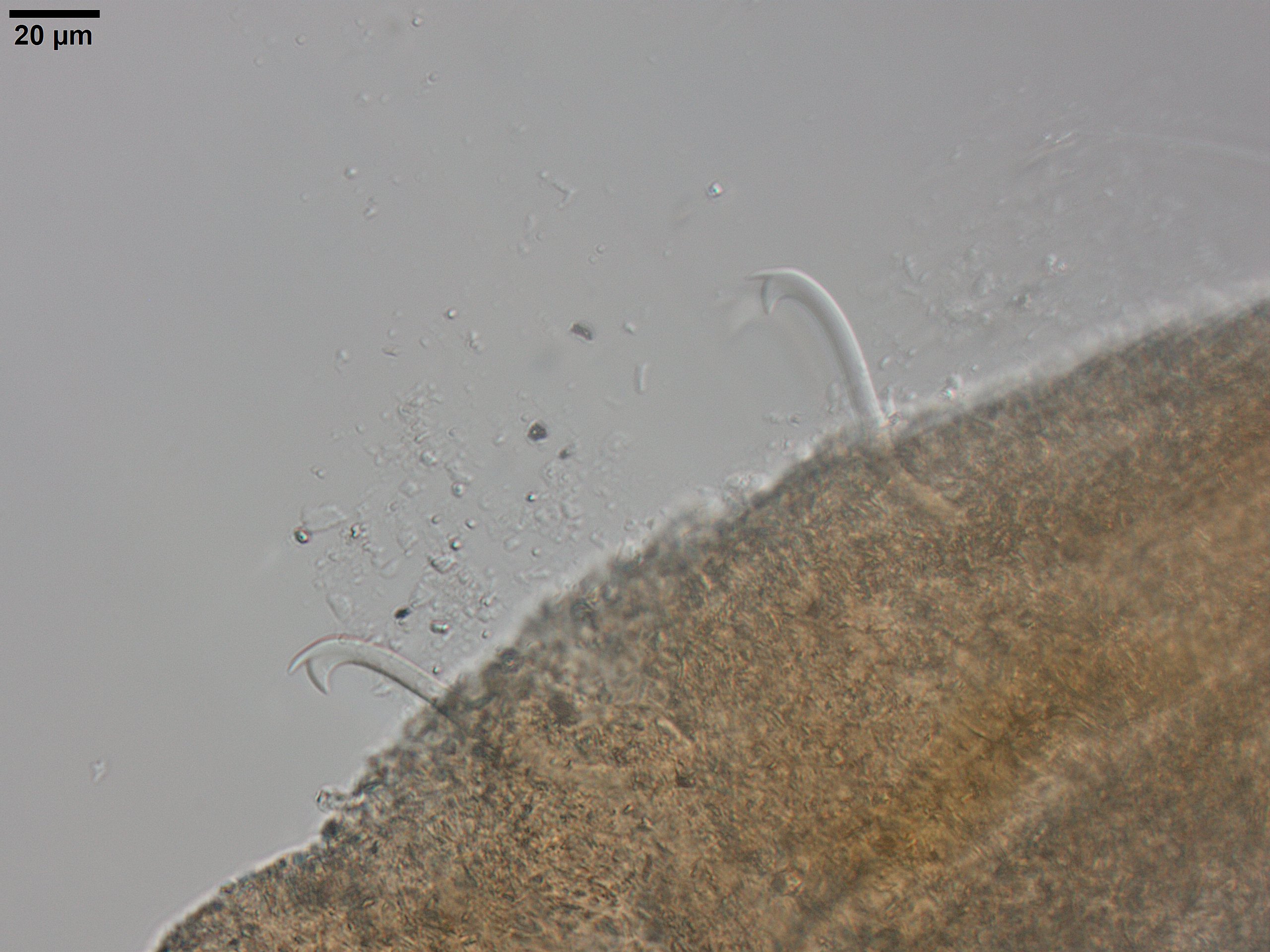

Supplement: Supplementary file 1 [file biology-09-00436-s001.zip › Supplementary_Figure_S4.jpg]

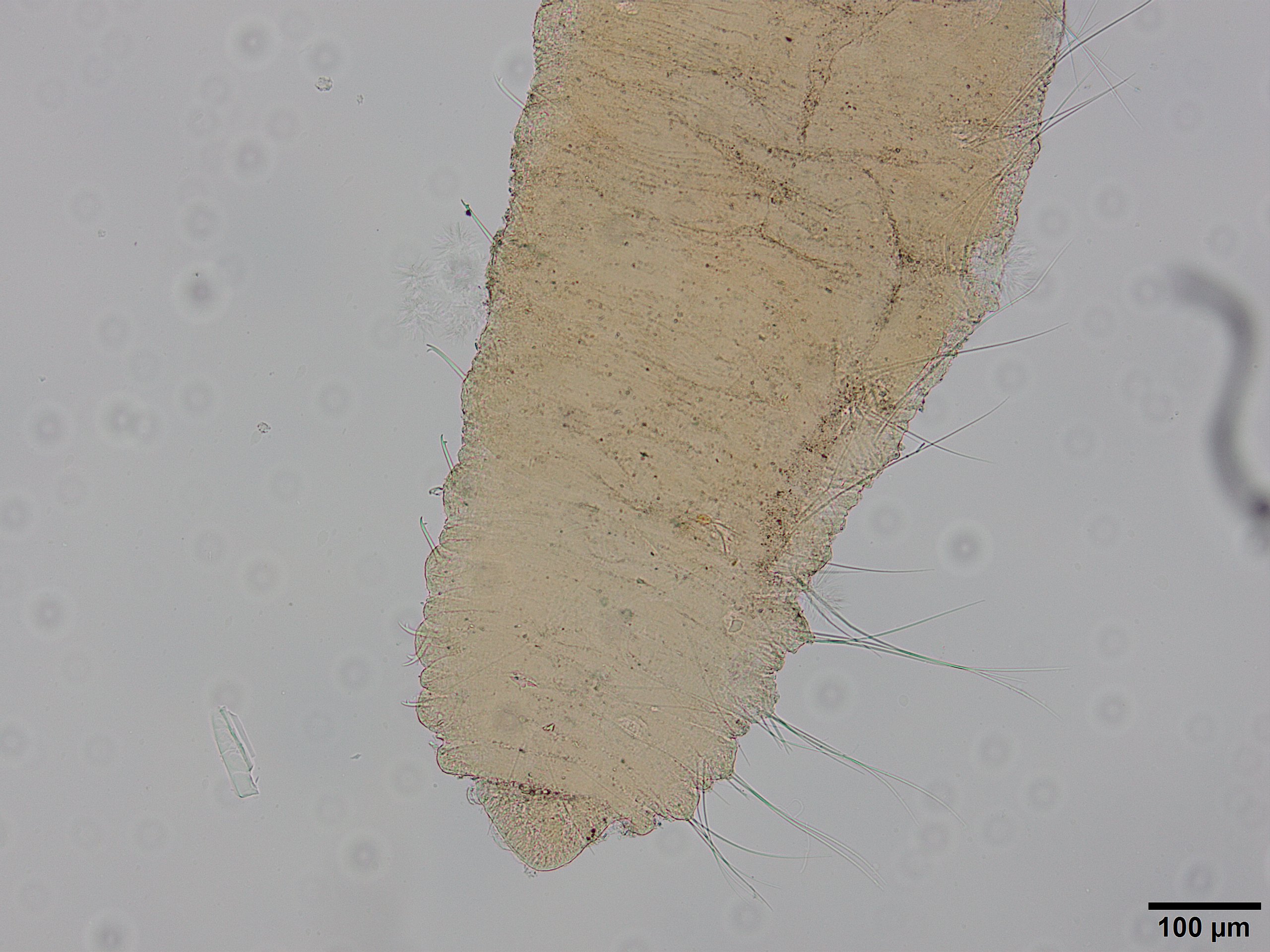

Supplement: Supplementary file 1 [file biology-09-00436-s001.zip › Supplementary_Figure_S5.jpg]

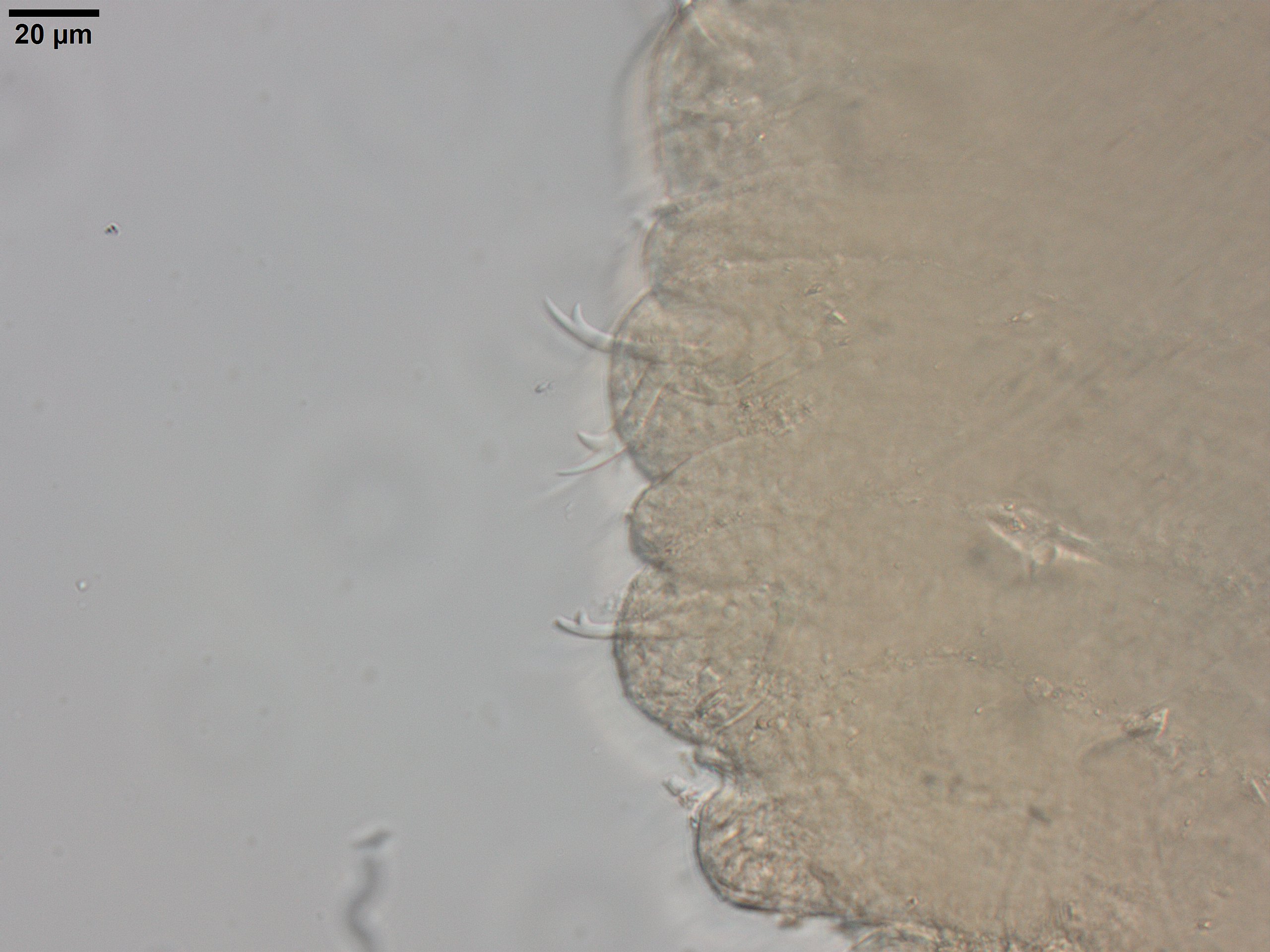

Supplement: Supplementary file 1 [file biology-09-00436-s001.zip › Supplementary_Figure_S6.jpg]

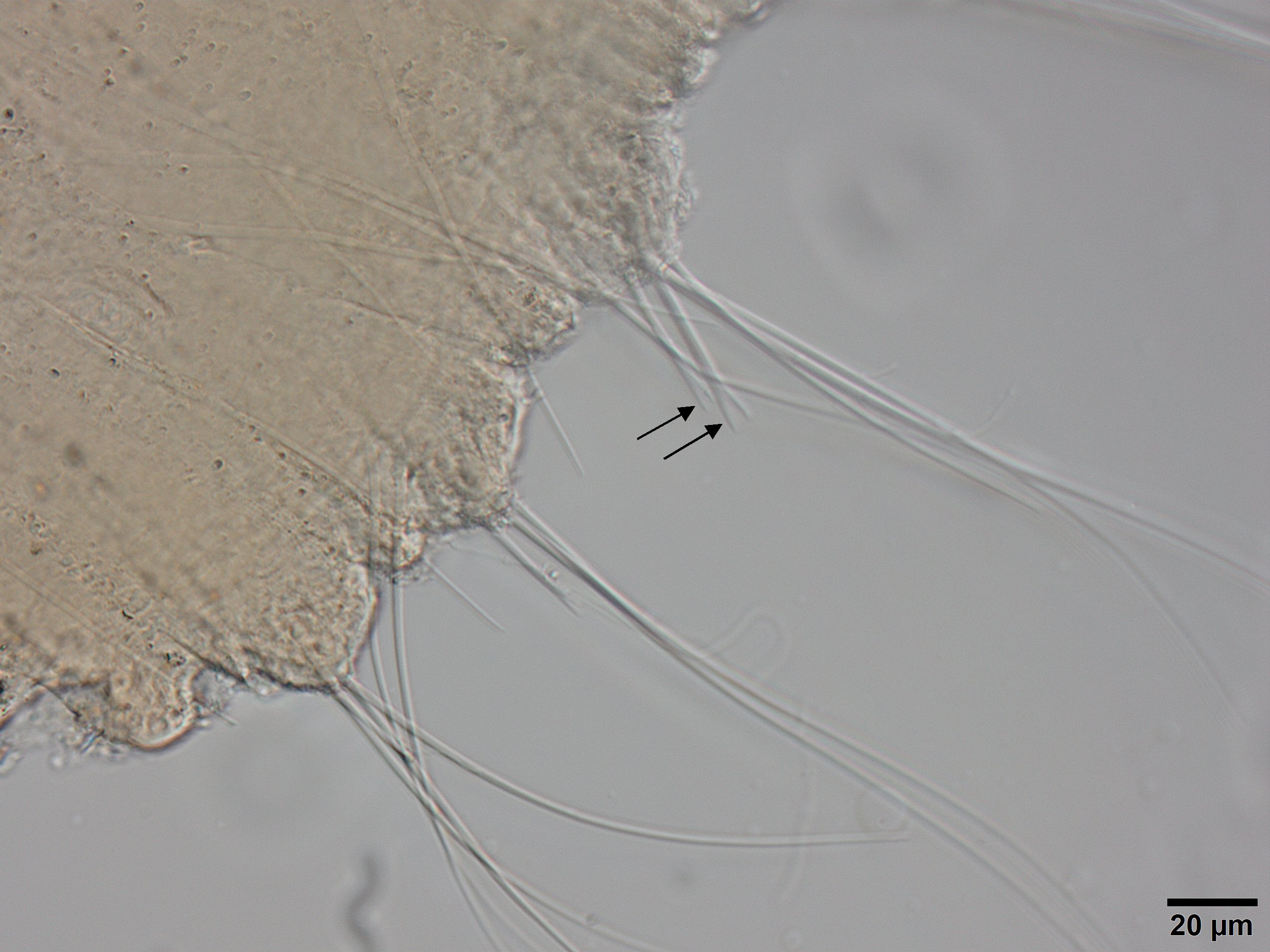

Supplement: Supplementary file 1 [file biology-09-00436-s001.zip › Supplementary_Figure_S7.jpg]

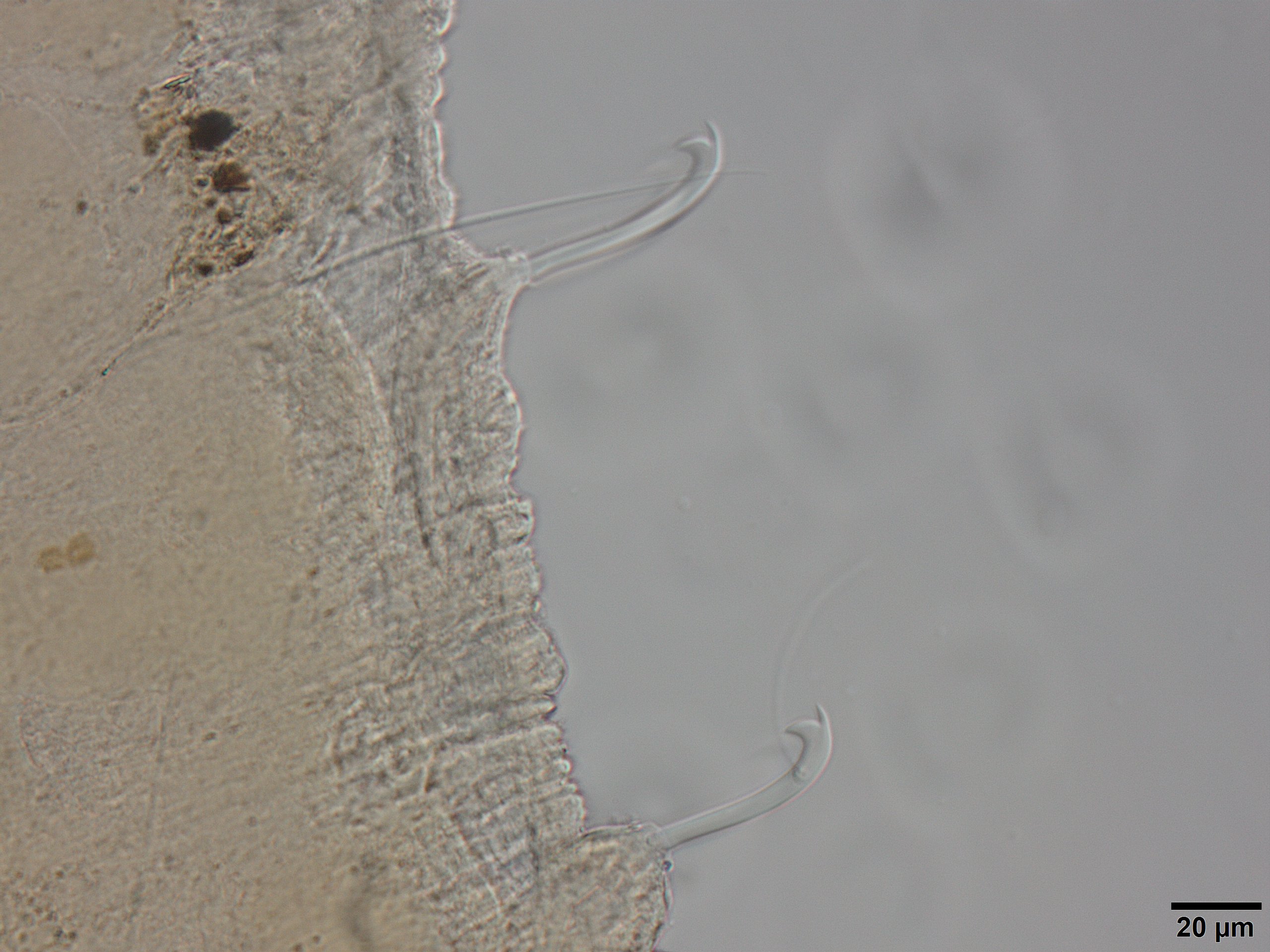

Supplement: Supplementary file 1 [file biology-09-00436-s001.zip › Supplementary_Figure_S8.jpg]

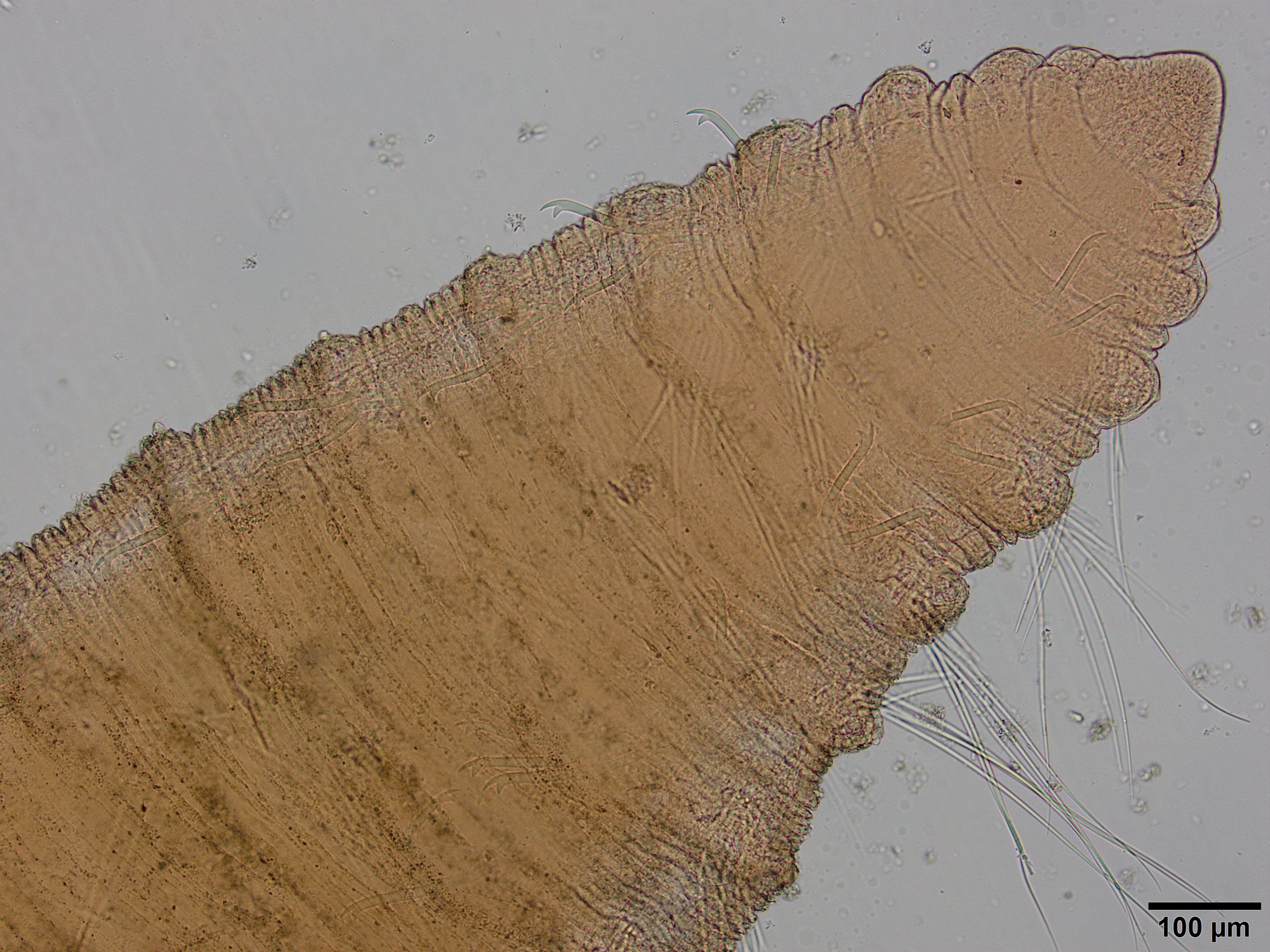

Supplement: Supplementary file 1 [file biology-09-00436-s001.zip › Supplementary_Figure_S9.jpg]
